# Planting Seeds for the Future: Scoping Review of Child Health Promotion Apps for Parents

**DOI:** 10.2196/39929

**Published:** 2023-07-20

**Authors:** Sarah B Blakeslee, Kristin Vieler, Ingo Horak, Wiebke Stritter, Georg Seifert

**Affiliations:** 1 Department of Pediatrics Division of Oncology and Hematology Charité - Universitätsmedizin Berlin Berlin Germany; 2 Digital Health Entrepreneur Berlin Germany

**Keywords:** scoping review, child health promotion, parents, mobile apps, health apps, digital prevention, behavior change, mHealth

## Abstract

**Background:**

Increasingly, parents use child health promotion apps to find health information. An overview of child health promotion apps for parents currently does not exist. The scope of child health topics addressed by parent apps is thus needed, including how they are evaluated.

**Objective:**

This scoping review aims to describe existing reported mobile health (mHealth) parent apps of middle- to high-income countries that promote child health. The focus centers on apps developed in the last 5 years, showing how the reported apps are evaluated, and listing reported outcomes found.

**Methods:**

A scoping review was conducted according to PRISMA-ScR (Preferred Reporting Items for Systematic Reviews and Meta-Analyses for Scoping Reviews) guidelines to identify parent apps or web-based programs on child health promotion published between January 2016 and June 2021 in 5 databases: PubMed, ERIC, IEEE Xplore, Web of Science, and Google Scholar. Separate sources were sought through an expert network. Included studies were summarized and analyzed through a systematic and descriptive content analysis, including keywords, year of publication, country of origin, aims/purpose, study population/sample size, intervention type, methodology/method(s), broad topic(s), evaluation, and study outcomes.

**Results:**

In total, 39 studies met the inclusion criteria from 1040 database and 60 expert-identified studies. Keywords reflected the health topics and app foci. About 64% (25/39) of included studies were published after 2019 and most stemmed from the United States, Australian, and European-based research. Studies aimed to review or evaluate apps or conducted app-based study interventions. The number of participants ranged from 7 to 1200. Quantitative and qualitative methods were used. Interventions included 28 primary studies, 6 app feasibility studies, and 5 app or literature reviews. Eight separate topics were found: parental feeding and nutrition, physical activity, maternal-child health, parent-child health, healthy environment, dental health, mental health, and sleep. Study intervention evaluations cited behavior change theories in 26 studies and evaluations were carried out with a variety of topic-specific, adapted, self-developed, or validated questionnaires and evaluation tools. To evaluate apps, user input and qualitative evaluations were often combined with surveys and frequently rated with the Mobile App Rating Scale. Outcomes reported some positive effects, while several intervention studies saw no effect at all. Effectively evaluating changes in behavior through apps, recruiting target groups, and retaining app engagement were challenges cited.

**Conclusions:**

New parents are a key target group for child health apps, but evaluating child health promotion apps remains a challenge. Whether tailored to parent needs or adapted to the specific topic, apps should be rooted in a transparent theoretical groundwork. Applicable lessons for parent apps from existing research are to tailor app content, include intuitive and adaptive features, and embed well-founded parameters for long-term effect evaluation on child health promotion.

## Introduction

Digital health is a growing field and apps are used regularly to target health prevention. eHealth measures have steadily gained popularity and are increasingly available in the app form. For the promotion and maintenance of health, digital interventions have been examined for their ability to work as a preventive measure [1]. An increasing number of apps target parents and children for child health promotion and well-being, yet little is known about their impact. Research is conclusive that health promotion activities for child health have a long-term impact on health, whether it be mental health, physical activity, nutrition, or risk behavior prevention [2-6]. Smartphones are estimated to be owned by over 50% of the world’s population (~4.3 billion people by 2023) [7], with smartphone ownership averaging over 75% in countries with high-level economies such as the United States and the European Union [8]. Nearly all adults (96%) aged 18-29 own a smartphone in the United States [9] and in Europe on average 75% of people in this age bracket use the internet every day [10]. Current parents and the next generation of parents are seeking health information from digital sources and increasingly from apps, demonstrating the opportunity for health promotion through app use [11].

Stemming from different theoretical approaches from health psychology and fields studying social behavior [12-16], a need to evaluate the ability of illness prevention and health promotion interventions to change behaviors led to the development of behavior change techniques (BCTs) [17]. These are categories of evaluable information, termed taxonomies, that track and measure how effective health promotion interventions can be [18]. The application of such evaluative measures in digital interventions has become a well-established method to evaluate changes in behavior over the last decade [19,20]. For instance, there has been some evidence demonstrating moderate effects of health apps on physical activity and diet in pregnant women [17,21], adults [22], or children [23]. A recent meta-analysis of apps directed at health promotion and illness management described the need for stronger evidence to underscore their effects [24]. At the same time, when it comes to the promotion of health, not enough is known about how or if the use of apps has an effect on behavior change, nor to what extent the evaluation of such apps is undertaken [25], nor how this relates to the actual use of such health apps [26]. Despite the potential and opportunity for combining prevention activities into digital health apps, evaluation of behaviors to measure the effectiveness of mobile interventions is imperative to demonstrate any impact on well-being.

New parents bestow both the genetic makeup and the preliminary foundation for health to their children—from pregnancy to independent adulthood. Despite being an essential cornerstone and stakeholder of child health promotion and well-being, parents often feel unprepared for parenthood [27] and ill-informed about their child’s development [28]. There has been no review to our knowledge that assesses if and how child health promotion broadly targeted in parent-based interventions is being evaluated. In an ever-changing digital landscape with continually developed new apps, establishing what apps exist to target parenting and childhood health promotion as well as how they are evaluated is an area of interest.

A preliminary search of literature confirmed that reviews have systematically looked at the impact of apps on behavior [29], and also specific areas of health promotion have been systematically addressed for adults and children, such as nutrition or physical activity [17,30-32], literacy [33], pregnancy [32], and even general well-being [29]. However, a comprehensive compendium of apps that apply to parents for the health promotion activities in children does not exist nor are the evaluative effects of such apps clear. The need to better understand the scope of what apps exist and how they are currently evaluated provides the rationale for this review. The aim of this scoping review is therefore to address this gap by reviewing the existing studies on mobile health (mHealth) prevention apps that target parents for promoting the health of their children. The primary objective of this review is to describe existing reported mHealth parent apps of middle- to high-income countries that promote child health, with a focus on the parent apps developed in the last 5 years. To achieve the objective, this paper intends to give an overview and details on the topic areas of health promotion that parent apps cover and presents the scope of apps that are reported on (keywords, year of publication, country of origin, aims/purpose, study population and sample size, intervention type, and methods). The secondary objective of this review is to compile a list of how the reported apps are evaluated by listing and describing health measures found. The research questions that guided this review were as follows: What current parent mHealth apps exist in middle- to upper-income countries for promoting child health and how, when, and where are they reported on? What topics do they cover? How are child promotion apps for parents evaluated and what outcomes are described in terms of their effectiveness and efficacy? This scoping review aims to shed light on and give a comprehensively reported overview of existing parent apps to promote children’s health.

## Methods

### Design and Overview

A scoping review method was chosen as the appropriate review type to give a broad overview of the existing apps on child health available for parents because this field has not yet been comprehensively mapped and ever-emerging evidence rapidly changes. A planned 3-step search strategy study protocol was registered with the Open Science Forum [34] and used with an established scoping framework [35-37] to search for apps geared toward parents for health promotion in children. The scoping review reporting was supported throughout by the PRISMA-ScR (Preferred Reporting Items for Systematic Reviews and Meta-Analyses for Scoping Reviews) checklist [38].

### Parental Mobile App Study Search Strategy

In a first step, from May 26, 2021 to May 28, 2021, 4 available databases were searched in 2 rounds to include the fields of health, education, and technology: PubMed, ERIC, IEEE Xplore, and Web of Science. After the first-round search with Google Scholar (Google Inc.), too many undifferentiated resources outside the inclusion were found for the search terms, and thus we decided to strategically limit the search to 2021 to find the most recent publications that may be found in the first months after publication, but before these are added to other databases. Search terms combined the keywords “health promotion,” “parent*,” “child*,” and “app,” “eHealth” and “mHealth,” “mobile health prevention,” and “digital health” (Multimedia Appendix 1). Inclusion and exclusion were described and then tailored after the initial search with the study team (SBB, WS, IH, and GS).

In a parallel organizational step to include health expert input from May to August.20, 2021, the third author (IH), gathered stakeholder inputs with authors and health experts located in Germany and Europe to identify parenting studies or apps that may not have been included. This was conducted first through a LinkedIn (Microsoft Corporation) post from a well-established networking account asking for expert input(s) on apps or research projects aimed at young parents to promote the health of their children from birth and how these have been assessed or evaluated. From the expert responses, this information was followed up on to elicit more detailed information on known apps.

### Eligibility and Exclusion Criteria

**Figure 1:**
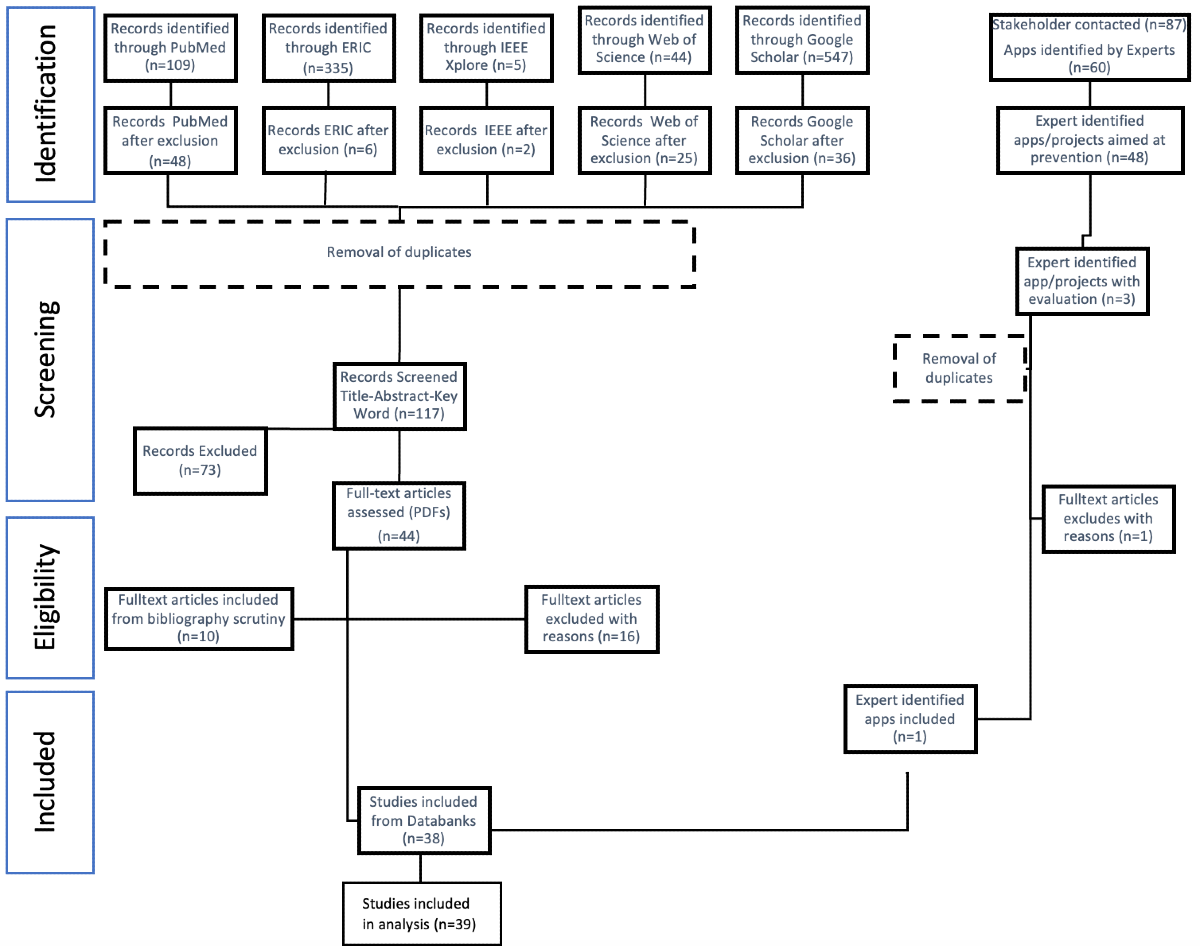
PRISMA-ScR flowchart. PRISMA-ScR: Preferred Reporting Items for Systematic Reviews and Meta-Analyses for Scoping Reviews.

Apps or projects that met the inclusion criteria (Table 1) were assessed further. Study inclusion and exclusion were documented at each step (Figure 1). We aimed to include studies, evaluations, and assessments of digital apps developed toward parents for child health promotion. Studies of all types, reports, and assessments were included if they were (1) digital apps (2) used primarily by parents or expectant parents for (3) health promotion of children without a diagnosis or risk.

We included both primary studies and reviews of studies and apps. Gray literature was included as long as there was an evaluative component to the work. The apps could be web or mobile-based programs. Based on content, we allowed for a broad range of study interest as it applied to both programs and the people these programs were applied to, including app feasibility or design, evaluation of the apps themselves, evaluation of the potential or actual effect on behaviors, or discussed evaluation strategies. For the expert input, we included studies collected from German or European digital health experts, child health experts, educational experts, or study authors. Only studies based in a middle- or high-income country and published in or after 2016 were included because we were particularly interested in the most recent apps and contexts most resembling the German context of our own research.

All studies that aimed to manage illness or high risk of illness were excluded. Exclusion was applied to any apps or programs aimed solely toward professions or children or where parents were simply gatekeepers. Additionally, studies on apps that were only used as health monitoring, tracking, product-based devices, or as information communication tools such as for text messaging/SMS transmission, videoconferencing, or telehealth were removed from review.

**Table 1:** Inclusion and exclusion criteria overview.

Selection category	Inclusion criteria	Exclusion criteria
Study population	Expectant parents, parents, parents and children together	Professionals use in work setting, primary use by children with parents only as an app gatekeeper
Health area	All areas of illness prevention/health promotion	Apps for active management of diagnosis, illness, secondary disease prevention, sexual health, those that are institution based, or those recruiting high-risk patients
App type	Smartphone/tablet/desktop	Telehealth, text messaging/SMS-based health support, videoconferencing, health product–based, app only for tracking device facilitation, virtual reality
Publication type	Empirical studies, reports, reviews, study synthesis, meta-analysis, theses, study protocols	Guidelines, handbooks, instructional manuals, user-based information, technical or specialist publications, commentary, product description
Content of interest	App design, reports on app functionality, evaluations of apps and study reviews, behavior change techniques reporting or evaluation, evaluation strategies, structured digital application	Review of app functionality, usability survey results
Countries of interest	All upper-middle or high-income country context [39]	≤Lower- to middle-income country contexts
Stakeholder input	Digital health experts, child health experts, educational experts, study authors (focus on Germany and Europe)	No restrictions applicable
Timeframe	≥2016 (Google Scholar >2021)	<2015

### Study Selection

The search took place following an initial identification of studies through the databases. Then, we performed a screening of the title, abstract, and keywords for applicability according to the inclusion and exclusion criteria and studies were imported into EndNote X9 (Clarivate) [40].

In the next screening step, the first author (SBB) applied the inclusion and exclusion criteria according to study abstracts, eliminated duplicates, and added full-text PDFs of all studies fitting the inclusion criteria. All expert contributions were controlled for documentary evaluation or assessment of the apps or projects, ensuring they fit within the inclusion/exclusion criteria and removing duplication. The resulting full-text studies and corresponding research information system (RIS) files that compiled bibliographic data information were imported into the analysis management software MAXQDA (version 20; VERBI GmbH) [41].

All studies that passed the original screening were reviewed in full text, coded deductively with the bibliographic RIS content, and systematically evaluated according to the paper sections. After full-text scrutiny, studies not meeting the inclusion criteria were excluded and adjustments were discussed, justified, and made within the whole team when necessary, based on the refinement of the inclusion criteria. Additionally, scrutiny of the included bibliographies, especially topically relevant reviews, was culled for additional studies.

### Summarizing the Data

The included studies summarized the key information as suggested by Peters et al [35] and this key information was analyzed through a systematic and descriptive content analysis based on Mayring and Fenzl [42] using a combined deductive and inductive approach. Deductive coding and descriptive analysis were conducted on all the included studies to compile and describe the following information: (1) keywords, (2) year of publication, (3) country of origin, (4) aims/purpose, (5) study population and sample size, (6) intervention type, (7) methodology/method(s), (8) broad topic(s), (9) evaluation, and (10) outcomes and details. Following this, key findings that related to the scoping review questions were coded inductively within the deductive descriptive categories: broad paper topics and evaluation. To verify the reliability of the coding of the study types and topics, the second author (KV) reviewed all studies based on inductively developed definitions. Discrepancies were discussed within the team and code definitions were adjusted accordingly. A descriptive summary of how apps and behaviors were evaluated are summarized in Table 2.

**Table 2:** List of evaluation tools found in included studies.

Broad paper topics and evaluation tool category			Evaluation tool type or name [reference]			
**Physical activity**			Assessment of subcategories: changes in physical activity, adult physical activity, family and social group physical activity, children’s physical activity evaluation, and tracking physical activity and real-time measurements			
	Moderators of physical activity	Ecological Momentary Assessment (EMA); Behavioral Regulation in Exercise Questionnaire; Self-Efficacy Scale; intention to participate in physical activity and to eat healthy foods [43] Barriers to Being Active Quiz; Self-Efficacy for Physical Activity Scale; Physical Activity Stages of Change [44]				
	Adult physical activity	Short Questionnaire to Assess Health-Enhancing Physical Activity (SQUASH) [45] International Physical Activity Questionnaire [43] Stanford Brief Physical Activity Survey [44] WHO^a^ physical activity criteria [46]				
	Family and social group physical activity	Modified National Board of Health and Welfare’s survey [47] Parental Support for Physical Activity Scale [48] The Social Support and Exercise Survey [44] Family Health Climate Scale [43] Family physical activity goal setting [47]				
	Children’s physical activity evaluation	Test of Gross Motor Development 2nd Edition (TGMD-2); The Burdette Outdoor Playtime Checklist [49]				
	Tracking physical activity	Average minutes per day physical activity [47] Accelerometers or pedometers [43,44,50] Physical Activity Diary [43]				
	Body measurements for physical activity	BMI or height and weight [43-45,47-50]				
**Parent feeding and nutrition**			Assessment of subcategories: food types and quality, parent feeding and food acceptance, food environment, food and body measurements, and breastfeeding			
	Food types and quality	Youth Risk Behavior Survey questions; The Behavioral Risk Factor Surveillance System Questions [51] The Willett Questionnaire Harvard Food Frequency; Healthy Eating Index (HEI) [45] Healthy Kids Survey [52] Food Frequency Questionnaire [43,45,48,53-55] Consumption of fruit, vegetables, water, soft drinks, and snacks [48]				
	Parent feeding and food acceptance	Infant Feeding Questionnaire [56,57] Parent Feeding Practices Scale; Child Feeding Questionnaire (CFQ) [57] Norwegian Mother and Child Cohort Study (MoBa) Questions; Children’s Eating Behaviour Questionnaire (CEBQ); Child Food Neophobia Scale (CFNS) [54] Infant Food Exposure and Parental Intentions to Offer Foods [56]				
	Food environment	The Family Eating and Activity Habits Questionnaire [50,57] Self-efficacy scales, food insecurity [52] Regulation of Eating Behavior Scale [43] Postpartum Partner Support Scale (PPSS) [58] Parenting Strategies for Eating and Activity Scale, Parenting Feeding Style Questionnaire [48] Parenting Practices Questionnaire, Parent Modelling Questionnaire, Family Support [57] Menu planning and shopping practices, healthy restaurant selection practices, family food preparation practices [51] Australian NOURISH study questionnaire [54] Environment and Policy Assessment Observation [59] School Food Checklist [60]				
	Food measurements	Fruit and Vegetable Intake Diary [43,53] 24-hour dietary recall of foods and beverages [57] Food photography and weighed food records [59,60] Caloric counting in kilojoules [59,60]				
	Body measurements for nutrition	Weight reporting [51] BMI or height and weight [45,47,48,50,51,54-56,61] Waist circumference [50,57]				
	Breastfeeding	WHO duration of exclusive breastfeeding, Breastfeeding Self-Efficacy Scale (BSES-SF) [58] Baby Eating Behaviour Questionnaire (BEBQ) [56]				
Dental health			Dental Knowledge Attitudes and Practices Questionnaire [62] Oral health behaviors in children and determinants of the Theory of Planned Behavior [63] Purposively sampled qualitative interviews [63]			
Sleep			Customized Sleep Profile (CSP); Brief Infant Sleep Questionnaire–Revised (BISQ-R) [64] Familial risk moderates the association between sleep and zBMI^b^; activity-based sleep-wake identification; Sleep Habits Questionnaire [57]			
Mental health			Center for Epidemiological Studies Depression Scale [44] Warwick-Edinburgh Mental Well-Being Scale (WEMWBS) [65] Edinburgh Postnatal Depression Scale (EPDS), State Trait Anxiety Inventory (STAI) [66]			
Parent child health			Patient activation measure (PAM); Functional, Communicative, Critical Health Literacy Scale [67] Patient Education Materials Assessment Tool (PEMAT) [68] The 21-item Asian Self-Identity Acculturation Scale [50]			
Healthy environment			Safety behaviors and behavioral intentions [69] Safety knowledge [69,70] Hot beverage scald risk and burn first-aid knowledge [71]			
Maternal health and parenting			Prenatal Interpersonal Processes of Care (PIPC) Scale [67] Maternal Self-Efficacy Scale [50] Pregnancy Discomfort Checklist [44] Parenting Self-Efficacy (Tool to Measure Parenting Self-Efficacy [TOPSE]) [65] Parenting Efficacy Scale, What Being a Parent of a Baby Is Like (WPBL), Perceived Social Support for Parenting (PSSP), Parent-to-Infant Bonding Questionnaire (PIBQ) [66]			
**App evaluation**			Assessment of subcategories: app quality, app usability, and app coverage			
	App quality			Suitability Assessment of Materials (SAM) [72] Persuasive System Design Model [73] Mobile App Rating Scale (MARS) [53,72,74] Semistructured and structured interviews [74,75] Participant app testing [74,75]		
	App usability			Just-in-time adaptive interventions [43] Push notifications [43,44,58] Gamification [43,46,58,71,74] Technology Acceptance Model (TAM) [76] Engagement Index Tool [75] The System Usability Scale (SUS) [74]		
	App coverage			Health-Related Website Evaluation Form: Developed Quantitative Tool for App Coverage [72]		

^a^WHO: World Health Organization.

^b^zBMI: sex- and age-standardized BMI.

### Collating, Summarizing, and Reporting Results

The analysis of keywords (1) was conducted from the bibliographic RIS data according to their frequency of appearance. Presentation of the overall findings from the deductive analysis of the study information 2-7 was summarized and detailed in Multimedia Appendix 2. Within the broad topic(s), ways apps and behaviors were evaluated and study-described outcomes 8-10 and details were analyzed, and then described and summarized in an iterative, inductive process used for the included studies, including a cross-reference between topics and evaluation tools listed within the studies (Table 2).

Reviews were included in this scoping review. For pragmatic organizational reasons, and because some of the primary source data did not fit the scope of our review objectives or fit our inclusion criteria, only the findings of the reviews themselves were included, not the primary literature that they were based on.

## Results

### Overview

Of the 39 studies included in this review of child health apps for parent use, most stemmed from US-, Australian-, and European-based research. A total of 8 overlapping health promotion topics that were addressed in 28 primary intervention studies, assessed in 6 app feasibility studies, and reviewed in 5 app or literature reviews were identified. The topics found in the inductive analysis were parental feeding and nutrition, physical activity, maternal-child health, parent-child health, healthy environment, dental health, mental health, and sleep. In primary intervention studies, behavior change theories were embedded in 26 studies and evaluations were carried out with a variety of topic-specific, adapted, self-developed, or validated questionnaires and evaluation tools. Methodologically, included studies were summarized and the effects, if any, of interventions were described. Reported study effects varied and used diverse tools to evaluate intervention effects. Alternatively, the feasibility of apps or health behaviors was assessed with a described combination of quantitative evaluation and survey tools along with user input. Included studies cited challenges in assessing healthy behaviors of children though parent apps, specifically in finding the appropriate way to evaluate changes in behavior through apps, recruiting target groups, and retaining app engagement.

Overall, 1040 studies from the 5 selected databases were analyzed and 60 apps and programs were gathered through the expert network. After screening for eligibility and duplication, and adding resources from reviews, 39 studies were included in total; 28 of these were found from databases, 10 were discovered by scrutinizing the bibliographies of included sources, and 1 resource was included from the expert input. An overview of study inclusion can be seen in the PRISMA-ScR flowchart (Figure 1).

### Keywords

Keywords of all included studies demonstrated the following terms according to the bibliographic RIS information from the studies. The 11 most frequently used keywords listed in 9 or more included publications (with listed frequency of appearance) were humans (n=19), female (n=14), child (n=13), health promotion (n=12), male (n=12), parents (n=12), mHealth (n=11), smartphone/s (n=10), mobile apps (n=10), adult (n=9) and infant/s (n=9; Multimedia Appendix 3).

### Year of Publication and Country of Origin

The included studies were published between 2016 and 2021, with two-thirds published between 2019 and 2021 and an uptick observed in 2019 (Multimedia Appendix 4). Among the upper-middle and high-income countries included, the majority came from the United States (n=15) [44,51,52,77-79], followed by Australia (n=13) [45,49,53,56,58-60,72-74,80-82] and then the European region (n=9) [43,46-48,54,55,57,83]. Included European countries with 1 study each were Belgium [48], the Netherlands [83], Portugal [57], Sweden [47], and the United Kingdom [83], with 2 studies each in Norway [54,55] and Germany [43,46]. Only 2 studies came from countries outside the global North (Singapore [66] and Iran [62]).

### Aim, Sample Size, and Intervention Type

Specific aims of the studies were diverse and ranged from creating a topic overview of existing studies or apps, assessing the feasibility of developed apps, to evaluating the effectiveness of a child health promoting intervention involving app or web-based content. There were 3 types of interventions that were included in our review: 28 primary studies [43-52,54-56,58-67,69,71,78,79,82], 6 app feasibility studies [70,73-75,83,84], and 5 reviews, of which 2 were literature reviews [53,57] and the remaining 3 were app reviews [68,72,85]. In the studies, the number of participants ranged from 7 to 1200. The review of apps included between 29 and 47 apps and the review of studies included 11 studies each. Methodologically, the studies were heterogenous in design and evaluation method. The clinical trial was the most frequent study design type for 21 studies [43-45,47-51,54-56,58-62,66,67,69,79,80] with most using the randomized controlled trial (n=15) and others with pilot, nonrandomization or experimental designs (n=6). Four of the included studies [43,47,59,69] published protocols of studies yet to be undertaken. The second most frequently undertaken type of evaluation was feasibility studies connected to the evaluation of app design features, testing, and functioning [53,70,73-75,83,84]. Quantitative and qualitative results were combined in the mixed method designs of 7 of the included primary (n=3) [63,65,70] and feasibility (n=4) [73,75,76,83] studies. A predominantly qualitative design was undertaken by 2 studies [63,84]. Of the 32 single studies, 25 individual project names were listed, of which 3 projects had 2 publications (Make Safe Happen [69,70], Swap It [59,60], and the Growing Healthy Program [56,76]) and 4 did not list a specific name [62,63,67,78]. An overview and summary of the included studies can be found in Multimedia Appendix 2.

### Broad Topics

The studies included could be sorted into 8 main prevention and child health promoting topics: parental feeding and nutrition (n=19) [43,45,47,48,51-53,55-61,72-74,82,86], physical activity (n=8) [43-49,84], maternal-child health (n=6) [44,45,65,67,75,85], parent-child health (n=5) [66,68,78,79,83], healthy environment (n=3) [69-71], dental health (n=2) [62,63], mental health (n=1) [66], and sleep (n=1) [64]. A crossover of these inductively derived topics occurred in some studies and these were not mutually exclusive; if a study descriptively included more than 1 topic, then the study was included in both topics. This occurred most frequently with studies that addressed parental feeding or nutrition and physical activity: this combination of topics was found for 7 of the studies. In 2 studies physical activity was addressed in combination with maternal health. Parental feeding and nutrition addressed nutritional intake for a range of ages: starting with nutrition in pregnancy [45]; feeding practices and nutrition for infants and young children, whether through breastfeeding or solid food [47,48,54-57,61,72,73,76]; or promotion of healthier school meals or family nutrition [43,51-53,59,60,74,87]. Included studies that broached physical activity were interested in either tracking the movement as part of the app-based intervention [44,45,47] or physical activity as part of obesity prevention, comprehensive child fitness, or overall family health [43,46,48-50,84]. All studies with a topical focus on maternal-child health targeted women in pregnancy. The parent-child health app studies included had an educative or informational focus on parenting and child health. Included apps promoting a healthy environment targeted home safety and accident prevention, while studies addressing dental health were concerned with caries prevention and dental hygiene. Mental health was addressed from the standpoint of overall child well-being and the sleep app studies included assessed the parent tracking of infant sleep schedules.

### Parent Mobile App Evaluation

#### Evaluation of Behavior Change in Apps

Many of the study evaluations assessed changes in intentions, knowledge, or behavior over time. In total, 26 studies listed at least one specific behavior change theory that the study evaluation was based on: Social Cognitive Theory was mentioned in 9 studies [44,47,48,51,54,55,58,66,79] and in 1 meta-analysis [57]; Self-efficacy Theory was mentioned in 3 studies [53,65,66]; Social Determination Theory also in 3 studies [43,48,67]; and the Behavior Change Wheel in 4 studies [46,59,61,74]. Some studies also used BCTs in their interventions (n=6) [43,47,57,59,78,85]. While most studies do not explicitly name the individual BCTs (n=20), 10 of these studies used BCTs. Among studies that mentioned techniques of behavior change, the most frequently cited were the BCT taxonomy by Michie et al [88], which was cited in 2 studies [47,57], and the mHealth theory–based taxonomy for mobile apps, which was also cited in 2 studies [78,85]. Individual BCTs mentioned in the included studies were shaping knowledge, identification of self as a role model, demonstration of the behavior, self-monitoring of behavior, self-belief, prompts/cues, goal setting (behavior and outcome), identity, and social support.

To measure the potential for change in behavior, multiple questionnaires were used that cut across topics. Some questionnaires that assessed changes in behavior were self-developed [51,54,55,85] or developed out of other validated questionnaires [48,59,62]. As an essential part of most behavior change models, the most frequently used validated questionnaires in the studies assessed self-efficacy as a predictor for changes in behavior for different topics such as motherhood, nutrition, breastfeeding, and physical activity. Measures for changes in self-efficacy or knowledge before and after the intervention were described to give an outlook for the continuation of the new behavior. Listed validated questionnaires used to evaluate behavior changes were the 10-item COM-B Self-Evaluation Survey (healthy family meals) [74], Maternal Self-Efficacy Scale (a 12-item scale measuring the mother’s self-efficacy for promoting healthy eating, physical activity, and in limiting noncore foods) [50], the 14-item short form Breastfeeding Self-Efficacy Scale [58] assessing breastfeeding confidence, Self-Efficacy for Physical Activity [44], the 10-item Parenting Efficacy Scale [66], and 36-item Parenting Self-Efficacy (Tool to Measure Parenting Self-Efficacy [TOPSE]) [65]. Increasing knowledge cut across topics, ranging from a healthy environment [69,70,80], physical activity or nutrition [47,52,54,78], dental health [62,63] parenting for health [50,65], or sleep [64,78]. Despite the objective to increase health knowledge of parents, not all studies undertook explicit evaluations to measure knowledge change.

Assessment tools were mentioned and used for specific topics. An entire overview of assessment tools for evaluating data and parameters can be found in Table 2.

#### Physical Activity

Physical activity was assessed through different means: 10 studies used physical activity measures [43-50,78,84]. We identified 21 separate measures that evaluated physical activity in 3 ways: specific behaviors as they related to quantified movement (ie, accelerometer), those that predicted or moderated the physical activity undertaken (ie, self-efficacy), and measures of the outcomes of physical activity (ie, BMI or weight over time). Of these tools, 17 used validated measures to assess physical activity. Wunsch et al [43] and Choi et al [44] measured the self-efficacy of physical activity specifically. Accelerometer to track steps and physical movement were used or planned in several studies [43,44,50]. BMI calculations were investigated in 6 studies [44,45,47-50] evaluating physical activity, especially when combined with the topic of nutrition and as a secondary parameter. In studies with small children, the evaluation measurements and intervention for physical activity were frequently given by the parents or primary caregivers. For instance, in the studies by Trost and Brookes [49] and De Lepeleere et al [48], the parental support for Physical Activity Scale was used. A strong connection of studies researching the topics of nutrition and physical activity demonstrated a crossover in evaluation tools used for body measurement, such as BMI calculated from height and weight [43,45,47,48,50]. Combined nutrition and physical activity likewise evaluated parent preferences within theory-guided domains for healthy goal setting [78].

#### Parent Feeding and Nutrition

In total, 20 studies [43,45,47,48,51-61,72-74,76,78] fell into the topic of parent feeding or nutrition and had the largest number of individual assessments. Overall, we were able to identify 41 assessment tools used in the studies that fit into 1 of 6 separate evaluative purposes (see as referenced in Table 2): measuring food amounts, taking body measurements for nutrition (often also for evaluating physical activity), assessing the ways and environment in which food is consumed, evaluating the quality of food consumed, examining parent feeding and young child food acceptance, or assessing breastfeeding-specific practice. Of the 41 assessment tools and questionnaires used, the majority (n=32) were validated tools. Six tools were self-developed specifically for the study and 3 further assessments were listed in the reviews and their origin was unclear. The Child Feeding Questionnaire was found to be the most frequently used questionnaire to assess parental feeding practices [50,54,57]. An instrument most frequently used for evaluating nutrition was the Food Frequency Questionnaire [43,48,54,55,57,59].

#### Dental Health

Four studies evaluated parameters of dental health. In the dental study by Zolfaghari et al [62], for instance, the authors used a self-developed questionnaire to assess parent knowledge and practices that combined the self-developed questions with other validated questionnaires [89-91]. A 24-item validated questionnaire designed by Van den Branden et al [92] to measure oral health behaviors in children and the Theory of Planned Behavior determinants was used, with permission, prior to and following use of the app [63].

#### Sleep

Only 1 study [64] specifically evaluated sleep as an mHealth intervention. This specifically assessed the sleep of infants and babies with a Brief Infant Sleep Questionnaire-Revised. However, an evaluation of the sex- and age-standardized BMI (zBMI) was found in Gomes et al’s [57] review of parental feeding practices and as part of a parent information needs assessment [78].

#### Mental Health

Mental health was assessed in 3 of the included studies [44,65,66]. The Warwick-Edinburgh Mental Well-Being Scale, a validated measure, was used by Deave et al [65], using a 14-item scale of subjective mental well-being and psychological functioning. Choi et al [44] used the Center for Epidemiological Studies Depression Scale to assess the mental health.

#### Parent Child Health

A total of 8 studies [50,66-68,71,78,79,83] were found to address parent-child health interactions, including the health of families, identity, and family-based evaluations. None of the evaluation tools broadly assessed the parent-child health interactions, but rather concentrated on the specific topic of interest for the parent-child interaction. For instance, Knowlden and Sharma [79] used the most general assessment. The authors developed separate evaluations of maternal-facilitated and child-behavior constructs based on Social Cognitive Theory to evaluate the parent-child health interaction [79] with an aim to address healthy child nutrition and physical activity. Other topic-oriented parent-child health parameters were also found that focused on evaluating educative [66-68,71,83] or identity parameters [50].

#### Healthy Environment

Three studies [69,70,80] specifically evaluated healthy environment through evaluations of safety behavior and first-aid knowledge.

#### Maternal Health and Parenting

Six studies [44,45,65,67,75,85] addressed evaluations of maternal health and 7 studies [48,65,66,68,75,78,79] looked at specific parenting parameters. In 1 study [65], the parenting self-efficacy was measured with the TOPSE. The TOPSE was used to compare mothers at 3 months after birth who had downloaded the Baby Buddy app with those who had not downloaded the app, controlling for confounding factors. The postnatal mental state was measured in Shorey et al [66] with a crossover of mental health and parenting and infant bonding tools.

#### App Feasibility (Quality and Usability)

The most frequent way by which child health apps for parents were assessed was through the Mobile App Rating Scale [53,72,74], developed by Stoyanov and colleagues [93]. To further assess the feasibility and quality of parent apps, a mixed methods approach was used for further development and contextual adaptation of feedback through interviews, where mostly semistructured interviews were conducted [73-75,83,84]. Qualitative assessments of the apps used in in-person, online, and telephone [73] semistructured interviews or focus groups were analyzed by a stated inductive or thematic analysis. Whereas app development approaches guided the qualitative interview data collection [73,75], explicit stating of the qualitative theoretical approaches for the interviews themselves was notably lacking in some studies [83,84]. Braun and Clark was the most frequently cited theoretical approach [70,74,75]. Furthermore, data analytic tools for coverage, usability, and engagement were used by several studies of apps [72,74-76]. Additionally, features of apps such as push notifications, gamification, and just-in-time adaptive interventions were used or listed for apps to retain engagement [43,44,46,58,71,74].

### Parent Mobile App Outcomes

#### Reported Evaluation Outcomes Based on Topics

The manner in which parent-based apps and interventions reported on outcomes in the primary studies was mixed. The study-reported effectiveness of an intervention was cited by many to depend on the length of the intervention, the intended intervention that was targeted, and whether an app included in-person support. Apps increasing knowledge seemed to be a particularly effective means to create a healthy environment with children [70,71] or to increase knowledge on child oral health [62]. An increase in physical activity of pregnant women was cited by 2 studies [44,50] and an 8-week app intervention was able to increase the physical activity performed by children, but this was not a significant outcome [49]. Increasing knowledge on nutrition was demonstrated in 1 study [52]; however, this intervention was coupled with in-person support classes. For nutrition outcomes, a reported increase in motivation or the consumption of fruit and vegetables in a child’s diet was reported by several studies [48,51,55] and healthier lunches saw less discretionary foods packed by parents who used an app [60]. Most improved outcomes with the interventions were not simply attributed to the use of the app alone, however. For example, a trial on dental hygiene demonstrated improvement for app users with a high level of perceived behavioral control, especially when coupled with regular dental checkups [63]. App-only outcomes demonstrated some positive effects for new parents of infants with sleep problems [64] and for improving parent bonding and self-efficacy after birth [66]. Outcomes in nutrition studies that relied on longer term growth outcomes saw little sustained or no positive effect over time with app use [54,56,61,79]. Indeed, studies on app-based interventions for baby food introduction and sustained healthy eating in early childhood highlighted the difficulty of achieving any sustained positive effect over time [54-56]. Across other topics, app support for partners of breastfeeding women or lifestyle advice for pregnant women resulted in no changed outcome with the apps and eHealth interventions [45,58], or even saw negative outcomes in the group receiving an app-supported intervention (ie, intervention group) to aid pregnant women decision-making [67]. This outcome supports a recommendation given in multiple interventions to use real-world interaction and support interventions in conjunction with the app [50,55,61,65,66]. Recruitment posed its own challenges. Particularly, in studies that aimed at healthier behaviors for children that were facilitated and necessitated parental support, authors employed several strategies: some recruited children but evaluated data from parents [59], some spoke of parent-child dyads [50,55,61], while others focused on the recruitment of families [51]. Some studies reported parents having higher education levels and potentially greater willingness to engage with the technology than a targeted population that would most benefit from the intervention [45,48,54,58,61,63,79].

#### App Evaluations of Behavior Changes and Parent Experience

A few studies highlighted the difficulty of customizing BCTs to their app content that combined the aims of the intervention with potential needs of parents and the ability to effectively evaluate these measures [56,65,78], a point that was discussed in additional detail in the reviews by both Gomes et al [57] and Biviji et al [85]. Particularly, the app reviews and a few studies underscored the gap of evidence-based apps with best practices among available apps for parents across health promotion topics [72,78,83]. Tracking of growth, pregnancy development, breastfeeding, dental hygiene, and diet were features that parents enjoyed, especially if these contents were tailored to the health parameters [53,63,77,83]. At the same time, features such as chat functions [53,73] or diaries [44] had mixed reviews or negative desirability by parents in the studies.

#### App Content Delivery and Technical Features

Keeping parents motivated to use the app was a challenge reported in multiple studies [45,56]. Other content delivery mechanisms, such as audio recordings (podcasts) [75] or videos [48], saw a high level of adherence in terms of the content consumption. Technical problems, interface challenges, or the inability to appropriately tailor app features were feedback highlighted by several studies [56,58,61]. The engagement with the apps by parents was described in a few studies to have the highest relevance for first-time parents [66,76] and retaining app or program engagement, particularly for the group targeted, was a challenge cited in multiple studies [46,56,61]. Features such as push notifications were seen as helpful delivery tools to maintain engagement with the app [44,58,60,61,76] and gamification was seen to have some success in achieving this goal [46,62,71]. Future designs for engaging parents reference increasingly developed “just-in-time” features to enhance practicability and interaction [43,74,76].

## Discussion

### Principal Findings

The 39 studies that met the inclusion criteria for this review reflected a wide range of child health topics: parental feeding and nutrition, physical activity, maternal-child health, parent-child health, healthy environment, dental health, mental health, and sleep. The 8 individual topics were concluded by an inductive analysis. Behavior change theories guided the research of 26 studies and topic-specific, adapted, self-developed, or validated questionnaires and evaluation tools were used to assess and report study outcomes. At the same time, challenges were reported in effectively evaluating changes in behavior through apps, recruiting target groups, and retaining app engagement.

An overall increase of publications on the topic may reflect the growing number of apps developed in general. The lower number of the published studies during 2020 may be an influence of the COVID-19 pandemic, a trend that we saw increase in a swift subsequent spot search in each of the included databases (see Multimedia Appendix 1). Since this review was conducted, 3 additional study results from included study protocols were published [94-96]. The demand and need for addressing child health promotion have only grown since the start of the COVID-19 pandemic [97] and digital mHealth solutions are forecasted to continue to grow [98]. The greater opportunity to digitally support child health through parents solidifies the need to make sure that parents have access to health promotion apps that are embedded in scientific evidence and best practices. Generally, the regulation of recruitment strategies was very bound to the study context and was a challenge highlighted by the studies in our findings. Varied descriptions of how potential participants were recruited and who was recruited detailed a level of complexity requiring consideration for study designs with multiple sites (homes and schools, for instance) and studied parties (children and parents).

Our findings highlighted the complexity of compiling evidence of behavior changes that are supported by apps and web-based programs for child health. When app interventions evaluated parents’ knowledge after use as a primary outcome, evaluation of the knowledge increase was easily assessed [52,62,69-71]. Evaluating the effectiveness of more complex interventions of health promotion as described in the included studies requires multiple evaluation tools and behavior-specific tailoring in order to see potential effects that may or may not continue in the long term. Prevention interventions in primary care with young children have been found to be exceptionally challenging to sustain over time, requiring complex interventions and involvement of multiple actors [99]. One additional impediment for long-term measurable changes could derive from the need for a clear theoretical underpinning and health mode within health promotion apps. With the absence of illness in the prevention setting, apps for health promotion could benefit from a health psychology theory–based development with a systematic evaluation in order to lead to substantial positive changes in behaviors [100]. The studies included in this review had varying degrees of theory embedded into the app design, which can provide a framework for evaluation. The most frequently used framework in the included studies was the Behavior Change Taxonomy [88] and its adapted version for mobile apps [20,101], which was itself developed from an expert collaboration. Many of the included studies were not transparent in reporting the link between the theory of behaviors and the evaluation parameters assessed or app features developed. On the whole, the multipronged strategies required for developing and evaluating apps for parents exhibit methodological agility and interdisciplinary collaboration. Interventions with demonstratable effectiveness were able to do this, as was markedly evident in the included studies compiled and reviewed on the topics of maternal child health, parent feeding, and lunch box nutrition [53,57,77].

Involvement of stakeholders is an imperative first step in the development of apps. Health experts bring expertise and scientific basis to the interventions for child health promotion and such expertise can be built on to further develop and adapt apps to changing evidence and circumstances. An example of this adaptation is the Growing Healthy program, where an initial study on childhood obesity prevention starting in infancy was published [61,102] and then compared in an upscaled study with another intervention [56] and followed by parent insights and feedback that were able to be integrated back into the app development in order to make them more intuitive and adaptive to specific engagement levels and identified target groups [76]. Parent feedback demonstrates that the apps are used most when the intuitive apps and features can address their needs and questions they have about their child’s health at the point when they need answers. While parents in the included studies were not always able to imagine what theoretical features would be useful [46,78], they provided strong feedback when asked for (for instance, [53,70,74,83,84]).

### Strengths and Limitations

This scoping review provides the first comprehensive overview of available mobile apps and web-based programs for use by parents aimed at the health promotion of their children. The 39 included studies were systematically categorized, provide a thorough summary of current evidence, describe some of the best practices for app development on this topic, and give a strong foundation for further research.

Despite this, this review is not without limitations. Inclusion criteria for this review were purposefully phrased broadly to be as inclusive as possible for apps aimed at parents. However, the multiplicity of study types was not foreseen and may have been more succinctly described. For instance, only including primary studies may have facilitated greater clarity in study summary. This methodological choice also hindered greater comparison between the studies. This study did not include an evaluation of outcomes, a step that would be helpful in future research to evaluate measured changes in behavior or effectiveness that the parent apps had. We also purposefully only included apps and programs from middle- and upper-income countries, apps that targeted healthy children without a diagnosis, and only studies published after 2015. This limitation may have therefore excluded apps or programs in other contexts that may have had broader and more global application. A future review would benefit from a systematic evaluation of app outcomes that includes only primary studies with inclusion of middle- and lower-income countries to be more generalizable and relevant to a larger population. Despite our attempts to include potential gray literature and expert input, no unpublished app evaluations were found. Despite our best efforts to include studies from other disciplines, most apps for parents, which were aimed at the health of their children, were found and evaluated within the health field. Access to published analysis of apps with detailed information evaluation is likely a further limitation of this study, because of the assumption that most apps developed in a scientific context are motivated to publish on the development and evaluation findings. It must be recognized that apps are developed out of many contexts and future reviews would benefit from the inclusion of parent apps developed from other fields (eg, marketing, industry, governmental or nongovernmental organizations, or other interest groups). Our own attempt to bridge this gap with the addition of extending and tapping into an expert network only saw limited methodological success.

### Conclusions

Existing apps and web-based programs aimed at parents to promote the health of their children cover a broad range of topics. Most aim to modify the nutrition and physical activity behavior—important for lifelong prevention of illness. New parents are a key target group for apps, whether to increase their knowledge or parental self-efficacy. Evaluating apps for child health promotion provides a special challenge and must be tailored to the needs of parents, context of the topic, and are ideally rooted in a transparent theoretical framework. Given the increasing digitalization of health and expanding focus of health policy on prevention measures, parent apps are guaranteed a role in our lives. Lessons learned can be garnered from existing research studies that tailor developed content to target group needs, include intuitive and adaptive features, and embed well-founded parameters for evaluations able to investigate long-term effects of parent apps on child health.

## Appendix

Multimedia Appendix 1 Search strategy details.

Multimedia Appendix 2 Detailed summary of results.

Multimedia Appendix 3 Research information system (RIS) keywords from all included studies (occurrence in publications ≥2).

Multimedia Appendix 4 Included scoping review publications (2016-2021).

## Abbreviations

MARS: Mobile App Rating Scale
mHealth: mobile health
PES: Tool to Measure Parenting Self-Efficacy
PRISMA-ScR: Preferred Reporting Items for Systematic Reviews and Meta-Analyses for Scoping Reviews
RIS: research information system
zBMI: sex- and age-standardized BMI
