# Supplementary material for: Planting Seeds for the Future: Scoping Review of Child Health Promotion Apps for Parents
Source: JMIR Mhealth Uhealth. 2023 Jul 20;11:e39929. doi: 10.2196/39929 (PMC10401193; doi:10.2196/39929)
Supplement: Multimedia Appendix 1 [file mhealth_v11i1e39929_app1.docx]

Multimedia Appendix 1: Search Strategy Scoping Review

| Search Round 1 |  |  |  |  |  |
| --- | --- | --- | --- | --- | --- |
| Term Search <Round 1> | PubMed (May 27, 2021) | ERIC (May 20, 2021) | IEEE Xplore (May 28, 2021) | Web of Science (May 18, 2021) | Google Scholar (May 20, 2021) |
| Main Search Term for all Databases:   - Health - Promotion - App child*   Additional terms added to main terms:  mobile   - mhealth - application* - mobile application* | - 2016-2021 - Health promotion App - Health promotion apps - Child Health promotion applications - Child health promotion mobile applications   Child health promotion mhealth | - Last 5 years button (>2017) - Health promotion App - Health promotion apps - Child Health promotion applications - Child health promotion mobile applications | - 2016-2021 - Health promotion App - Health promotion apps - Child Health promotion applications - Child health promotion mobile applications - Child health promotion mhealth   Child health promotion parents | - Publication Date range: 2016-2021 - Health promotion App - Health promotion apps - Child Health promotion applications   Child health promotion mobile applications | - Specified Publication Date range: 2016-2021 - Health promotion App - Health promotion apps - Child Health promotion applications - Child health promotion mobile applications - Health promotion App parent* - Health promotion apps parent* - Child Health promotion applications parent* - Child health promotion mobile applications parent*   Publication date for search strategy |
| Search Round 2 |  |  |  |  |  |
| <Round 2> | Pubmed (May 28, 2021) | ERIC (May 28, 2021) | IEEE Xplore | Web of Science  (May 20, 2021) | Google Scholar (May 27, 2021) |
| Addition of parent descriptions in searches | - 2016-2021 - Health promotion App - Health promotion apps - Child Health promotion parent* - Child health promotion app* parent   Child health promotion parent* mhealth | - Descriptor: Parents - Last 5 years button (>2017) - Health promotion App - Health promotion apps - Child Health promotion applications - Child health promotion mobile applications | No second search was done after very few publications were found in first search | - Publication Date range: 2016-2021 - Health promotion App - Health promotion apps - Child Health promotion applications   Child health promotion mobile applications | (using ≥2021 to limit number)  “Health promotion”+ mobile “App” + parent child* |
| Subsequent Search 2023 | Number of publications for each database over years 2021-2023  searched on 27 March 2023 | | | | |
| Publish Year: 2021-2023 | Pubmed  Searched: "Health Promotion" App child* | ERIC | IEEE Xplore | Web of Science  Searched: Child health promotion mobile applications | Google Scholar  Searched:  "Health Promotion" App child* |
| 2021 | 21 | 1 | 0 | 20 | 3800 |
| 2022 | 31 | 40 | 1 | 18 | 11720 |
| 2023 | 21 | 27 | 0 | 1 | 1670 |
