# Supplementary material for: Planting Seeds for the Future: Scoping Review of Child Health Promotion Apps for Parents
Source: JMIR Mhealth Uhealth. 2023 Jul 20;11:e39929. doi: 10.2196/39929 (PMC10401193; doi:10.2196/39929)
Supplement: Multimedia Appendix 2 [file mhealth_v11i1e39929_app2.docx]

*Multimedia Appendix 2: Detailed Summary of Results*

| **Author (year)/ Country/ Reference** | **Study Project Name/ Intervention Type** | **Number of Participants (P)/**  **Apps (A)/ Studies (S)** | **Study population and recruitment/ included Apps/ included Studies** | **Objective** | **Methodology** | **Main Results** |
| --- | --- | --- | --- | --- | --- | --- |
| Au et al. (2016)/ USA[1] | Supplemental nutrition program for Womans, Infants and Children (WIC)/ Primary Study | 590 (P) | Mothers with children, recruited from supplemental nutrition program | **Objective**: Study changes in knowledge, attitudes, and behaviors of parents online vs. in-person group of breakfast nutrition education. | **Design**: Randomized-controlled trial comparing the effectiveness of online and in-person nutrition education.  **Primary parameter:** questionnaire at baseline and a 2- to 4-month follow-up to  assess breakfast knowledge, attitudes, and behaviors. | **Primary finding**: Changes in knowledge between pretest and follow-up at 2 to 4 months were similar between groups. Both groups reported reductions in barriers to eating breakfast. Increases in the frequency of eating breakfast were greater for both the parent (P=0.0007) and child (P=0.01) in the online group compared with the in-person group. |
| Biviji et al. (2021)/ USA[2] | Literature/ App Review | 29 (A) | Apps, focus on maternal-child health, identified from Google Play and Apple Play Store | **Objective:** assess the presence of behavior change techniques (BCT’s) in popular maternal and infant health (MIH) mobile applications (apps) available in Apple app and Google Play stores. | **Design**: Identify and assess popular MIH apps for the presence of 16 BCTs using the mHealth app taxonomy.  **Primary parameter:** Included apps were downloaded in and coded for content analysis according to the BCT taxonomy. | **Primary finding**: .Twenty-nine apps could be included and coded. apps included seven BCT’s (range 2-16). Techniques such as personalization, review of general or specific goals, macro tailoring, self-monitoring of goals, and health behavior linkages were most frequently present. No differences in the presence of BCTs between paid and free apps were observed. Apps developed by healthcare developers incorporated a higher number of BCT’s. |
| **Author (year)/ Country/ Reference** | **Study Project Name/ Intervention Type** | **Number of Participants (P)/**  **Apps (A)/ Studies (S)** | **Study population and recruitment/ included Apps/ included Studies** | **Objective** | **Methodology** | **Main Results** |
| Burgess et al. (2018)/ Australia[3] | Cool Running/ Primary Study | 498 (P) | Australian women with at least 1 child aged 5-12 months. recruited via social media | **Objective**: to evaluate the efficacy of Cool Runnings, an app-based intervention to increase knowledge of childhood burn risk (specifically hot beverage scalds) and correct burn first aid among mothers of young children. | **Design**: 2-group, parallel, single-blinded randomized controlled trial (RCT). Both groups accessed an app throughout the study, but in the intervention group, gamification techniques were incorporated into the app.  **Primary parameter**:  change in knowledge about risk of burns and correct burn first aid at baseline and 6 months. | **Primary finding**: Intervention group participants achieved significantly greater improvement in overall knowledge posttest than control group participants. Significant correlations between gamification techniques and knowledge change (*P*<.001). Odds of knowledge improvement between baseline and 6-month follow-up was higher in participants with low-moderate app activity compared with no app activity and much higher in participants with high app activity. |
| Cheng et al. (2020)/ Australia[4] | Literature/ App Review | 47 (A) | Apps, focusing on parental feeding, included from Google Play and Apple Play Store | **Objective**: A review of existing child health apps to evaluate the quality of information on infant nutrition and physical activity. | **Design**: App evaluation on infant feeding, breastfeeding, formula feeding, and tummy time.  **Primary parameter**: App quality assessed using the 5-point Mobile App Rating Scale, readability, and suitability of health information. Quality of information on infant nutrition and physical activity was evaluated. | **Primary finding**: The information contained within the apps was poor: 64% of the evaluations found no or low coverage of the information found in the Australian guidelines on infant feeding and activity, and 53% of the evaluations found incomplete or incorrect information with regard to the depth of information provided. |
| **Author (year)/ Country/ Reference** | **Study Project Name/ Intervention Type** | **Number of Participants (P)/**  **Apps (A)/ Studies (S)** | **Study population and recruitment/ included Apps/ included Studies** | **Objective** | **Methodology** | **Main Results** |
| Choi et al. (2016)/ USA[5] | Fitbit Study/ Primary Study | 30 (P) | Pregnant women, recruited in public & private via flyer distribution | **Objective**: Feasibility of recruitment, randomization, and intervention and the potential efficacy of a 12-week mHealth physical activity program for physically inactive pregnant women. | **Design**: Pilot randomized controlled trial with two groups for gradual increases in physical activity during pregnancy with a 12 week mobile phone application and accelerometer vs. accelerometer only intervention.  **Primary parameter**: Weekly mean steps per day for the prior week, as measured by the accelerometer. | **Primary finding**: Intervention participants had a 1096 ± 1898 step increase in daily steps compared to an increase of 259 ± 1604 steps in control participants at 12 weeks. The change between groups in weekly mean steps per day during the 12-week study period was not statistically significant (p = 0.38). The intervention group reported lower perceived barrier to being active, lack of energy, than the control group at 12-week visit (p= 0.02). |
| Cullen et al. (2017)/ USA[6] | Family Eats/ Primary Study | 151 (P) | African-American families, recruitment at local community locations via flyers | **Objective**: Improving parent and child fruit and vegetable intake of African American families via an eight-session Family Eats web-based intervention. | **Design**: a two-group, randomized design with three data collection periods: baseline, 2 months after, and 4 months post intervention.  **Primary parameter**: Assessment of fruit and vegetable intake behaviors through questionnaires. | **Primary finding**: Significant positive changes overtime were noted for both groups for home fruit/vegetable availability, food preparation practices, and healthy restaurant selection. A reported a significant increase in home juice availability at Post 1 was found in the intervention group compared with the control group. Home fruit availability improved for both groups. |
| **Author (year)/ Country/ Reference** | **Study Project Name/ Intervention Type** | **Number of Participants (P)/**  **Apps (A)/ Studies (S)** | **Study population and recruitment/ included Apps/ included Studies** | **Objective** | **Methodology** | **Main Results** |
| Cushing et al. (2018)/ USA[7] | No Name/ Primary Study | 183 (P) | Parents using mhealth Apps, Recruitment over an online platform (Amazon MTurk) | **Objective**: to assess parent preferences for mobile app features that map onto specific Theoretical Domains Framework (TDF) | **Design**: A validity study of parents crowdsourced on a website who were asked to rate their preferences for mobile app features that correspond to elements of the TDF. The TDF organizes a large number of theoretical models and constructs into three components (1) capability, (2) motivation, and (3) opportunity.  **Primary parameter**: Survey questions targeted the importance of diet, physical activity and sleep and acceptability of these | **Primary finding**: Parents demonstrated a preference for increasing procedural knowledge for physical activity and diet behaviors over sleep (F_2,545_=5.18, P=.006)and self-monitoring as more important for physical activity than sleep (F_2,546_=4.04, P=.02). Asked about the features to help children develop skills, parents preferred features for dietary behavior over sleep (F_2,546_=3.57, P=.03), found goal-setting features for physical activity over sleep and diet (F_2,545_=5.30,  P=.005), and app incentive features for physical activity over sleep (F_2,546_=4.34, P=.01) most useful. |
|  |  |  |  |  |  |  |
| **Author (year)/ Country/ Reference** | **Study Project Name/ Intervention Type** | **Number of Participants (P)/**  **Apps (A)/ Studies (S)** | **Study population and recruitment/ included Apps/ included Studies** | **Objective** | **Methodology** | **Main Results** |
|  |  |  |  |  |  |  |
| Deave et al. (2019)/ UK[8] | The Bumps and BaBies Longitudinal Study (BaBBLeS)/ Primary Study | 488 (P) | Pregnant first time mothers, recruitment at five participating maternity units through invitation | **Objective**: Assessment of the effectiveness of the Baby Buddy app on improving maternal  self-efficacy and mental well-being at 3 months post-birth. | **Design**: Longitudinal, mixed methods study with three parts: a cohort study, analysis of in-app data and a qualitative study.  **Primary parameter**: Measure of Parenting Self-Efficacy (TOPSE) used to compare mothers at 3 months post-birth who had downloaded the Baby Buddy app with those who had not. | **Primary finding**: The Baby Buddy app did not elicit a statistically significant change in TOPSE scores from baseline to 3 months post-birth [adjusted odds ratio (OR) 1.12, 95% confidence interval (CI): 0.59 to 2.13, P=0.730]. No evidence for the effectiveness of the Baby Buddy app was found. |
| Dodd et al. (2017)/ Australia[9] | Smartphone nutrition and physical activity application to provide lifestyle advice to pregnant women (SNAPP)/ Primary Study | 162 (P) | Pregnant women with a body mass index ≥18.5kg/m^2^, with a singleton pregnancy between 10 and 20 weeks' gestation, and participating in 2 pregnancy nutrition‐based randomised trials. | **Objective**: Evaluation of the impact of a smartphone application as an adjunct to standard face‐to‐face consultations in facilitating dietary and physical activity change among pregnant women. | **Design**: Multicentre, nested randomised trial. Women were subsequently randomised to either the “Lifestyle Advice Only Group,” or the “Lifestyle Advice plus Smartphone Application Group”.  **Primary parameter**: The healthy eating index (HEI) was assessed by maternal food frequency questionnaire. | **Primary finding**: Mean difference in HEI score at 28 weeks of pregnancy was 0.01 (CI [−2.29, 2.62]) and at 36 weeks of pregnancy −1.16 (CI [−4.60, 2.28]). There was no significant additional benefit from the provision of the smartphone application in improving HEI score (p = .452). |
| **Author (year)/ Country/ Reference** | **Study Project Name/ Intervention Type** | **Number of Participants (P)/**  **Apps (A)/ Studies (S)** | **Study population and recruitment/ included Apps/ included Studies** | **Objective** | **Methodology** | **Main Results** |
| Gomes et al. (2021)/ Portugal[10] | Systematic review and meta-analysis | 12 (S)  (12 studies included in qualitative synthesis,  11 studies included in quantitative synthesis/  meta-analysis) | Studies with the topic of Parental feeding practices for parents of children 0-12 years, searched in 4 Databases (SCOPUS, Web of Science, EBSCO and CENTRAL) | **Objective**: Evaluation of randomized controlled trials of web-based interventions targeting feeding practices of parents of 0-12-year-old children, aiming to promote children's healthy diet or prevent nutrition-related problems. | **Design**: Systematic review and meta-analysis conducted from the earliest publication date until February 2020 to assess studies of parents of children between 0-12 years old with a randomized controlled trial intervention studies aimed to promote children’s healthy diet and/or to prevent nutrition-related problems through a web-based intervention with parental feeding practices.  **Primary parameter**: Meta-analysis and systematic review according to the Preferred Reporting Items for  Systematic Reviews and Meta-analysis (PRISMA) guidelines | **Primary finding**: Of the 1271 records found, 12 studies about nine programs were retained, comprising 1766 parents. Recent interventions, mainly directed to parents of young children were found, with small, non-clinical samples, and mostly theory-based. The programs were heterogeneous regarding the type of intervention delivered and its duration. The most assessed parental feeding practices were Restriction*,*Pressure to eat*,* and Food availability/accessibility*.* Meta-analysis indicated that most programs’ effects were small for the evaluated parental practices, except for Food availability/ accessibility that benefited the intervention group only when all follow-up measurements were considered. |
| **Author (year)/ Country/ Reference** | **Study Project Name/ Intervention Type** | **Number of Participants (P)/**  **Apps (A)/ Studies (S)** | **Study population and recruitment/ included Apps/ included Studies** | **Objective** | **Methodology** | **Main Results** |
| Helle (2019)/ Norway[11] | Early Food for Future Health/ Primary Study | 715 (P) | Parents with an infant 3-5 months, recruitment via Facebook and via email to child-health clinics | **Objective**: Evaluation of parental feeding practices to promote healthy child eating behaviors from infancy by measuring the increase of daily vegetable/fruit intake and promotion of more beneficial mealtime routines. | **Design**: two armed, randomized controlled intervention with a monthly age-appropriate video addressing infant feeding, corresponding cooking films/recipes, in the intervention group.  **Primary parameter**: child eating behaviors, dietary intake, mealtime routines and maternal feeding practices and feeding styles through questionnaires at 3 time points. | **Primary finding**: Fruit intake in the intervention group had a higher score 54.3% compared with of the control group at 48.3% (p = 0.29) and higher intake of vegetables in the intervention group 54.5% compared with 50.7% in the control group (p = 0.49). The children in the intervention group were also eating breakfast together with family ≥ 4 times per week at 65.7%, compared with 57.3% of the children in the control group (p = 0.12). |
| Henriksson et al. (2020)/ Sweden[12] | The MINISTOP 2.0 trial/ Study Protocol | 500 (P) | Families who attend a routine visit to one of 15-20 primary child health care centres throughout Sweden, when their child is 2.5 years | **Objective**: Evaluation of a parent-oriented mobile health app (the MINISTOP 2.0) integrated into primary child health care can improve diet and physical activity behaviors and reduce the prevalence of overweight and obesity in preschool-aged children | **Design**: a two-arm parallel groups randomized  controlled trial to investigate the effectiveness of the  MINISTOP 2.0 app on health behaviors, BMI, and parental self-efficacy with a 6-month parent-oriented smartphone intervention aimed at improving the dietary and activity behaviors and routine child health care for the control group at two time points.  **Primary parameter**: Dietary habits, physical activity and screen time while body weight and height in children, and parental self-efficacy are planned secondary outcomes | **Primary finding**: No results yet (study protocol). |
| **Author (year)/ Country/ Reference** | **Study Project Name/ Intervention Type** | **Number of Participants (P)/ Apps (A)/ Studies (S)** | **Study population and recruitment/ included Apps/ included Studies** | **Objective** | **Methodology** | **Main Results** |
|  |  |  | . | . |  |  |
| Knowlden & Sharma (2016)/ USA[13] | Enabling Mothers to Prevent Pediatric Obesity through Web-Based Education and Reciprocal Determinism (EMPOWER) / Primary Study | 57 (P) | Mothers with children 4-6 years, recruited at daycares and health care organizations via flyer | **Objective**: Evaluation of the efficacy of the EMPOWER intervention (Online, maternal based program for prevention of childhood obesity) at 1-year postintervention follow-up. | **Design**: Participant-blinded, equally randomized controlled parallel-group study. Mixed between-within subjects design evaluated across four time periods.  **Primary parameter**: change in maternal-facilitated social cognitive theory SCT construct scores (environment, emotional coping, expectations, self-control, and self-efficacy) as well as four child behaviors (physical activity, cups of fruits and vegetables consumed, sugar-sweetened beverages consumed, and screen time) measured at 3 time periods (4, 8, and 60 weeks). | **Primary finding**: A significant group-by-time interaction for child fruit and vegetable consumption was found in the experimental group (p = .012). At 1 year, results suggested an overall increase of 1.847 cups of fruits and vegetables (95% confidence interval = 1.207-2.498) in the experimental group (*p* < .001). Analysis suggested changes in the maternal-facilitated home environment accounted for 13.3% of the variance in the change in child fruit and vegetable consumption Improvements in child physical activity, sugar-free beverage intake, and screen time first detected at 1-month follow-up in both groups were no longer significant at 1-year follow-up. |
| **Author (year)/ Country/ Reference** | **Study Project Name/ Intervention Type** | **Number of Participants (P)/ Apps (A)/ Studies (S)** | **Study population and recruitment/ included Apps/ included Studies** | **Objective** | **Methodology** | **Main Results** |
|  |  |  |  |  |  |  |
| Laws et al. (2018)/ Australia[14] | Growing healthy & Baby’s First Food/  Primary Study | 645 (P) | Parent of an infant younger than 3 months or pregnant (30+ weeks gestation),  Recruitment via social media and online forums | **Objective**: to determine the feasibility and effectiveness of an mHealth obesity prevention intervention in terms of reach, acceptability, and impact on key infant feeding outcomes. | **Design**: A quasi-experimental study conducted with an mHealth intervention app and website containing information on infant feeding, sleep and settling, and general support for parents with infants aged 0 to 9 months and a nonrandomized comparison group at three time periods (baseline, 6, and 9 months of age).  **Primary parameter**: Assessment of breastfeeding duration and exclusivity, formula feeding, solid food introduction, child anthropometrics and app usage. | **Primary finding:** There were no significant differences between groups in any of the target behaviors.  Most parents (86.9%) downloaded and used the app; however, usage declined over time. There was a high level of satisfaction with the program, with 86.1% reporting that they trusted the information in the app and 84.6% claiming that they would recommend it to a friend. However, some technical problems were encountered with just over a quarter of parents reporting that the app failed to work at times. |
| **Author (year)/ Country/ Reference** | **Study Project Name/ Intervention Type** | **Number of Participants (P)/ Apps (A)/ Studies (S)** | **Study population and recruitment/ included Apps/ included Studies** | **Objective** | **Methodology** | **Main Results** |
|  |  |  |  |  |  |  |
| Ledford et al. (2017)/ USA[15] | System-Distributed Mobile Application in Maternity Care/ Primary Study | 205 (P) | Pregnant women attending antenatal care, hospital system recruitment at women and family medicine department & obstetrics | **Objective:** Testing the effectiveness of an app to support autonomous behavior and decision-making of pregnant women navigating prenatal care as compared to a distributed notebook. | **Design:** Randomized controlled trial conducted with pregnant woman navigating prenatal care in the Women’s Health Clinic and Family Health Clinic of three hospitals.  **Primary parameter**: Repeated-measures analysis of covariance was used to test intervention effects in the study sample. | **Primary finding**: Mothers used a mobile app interface to more frequently record information about their pregnancy; however, across time, mothers using a mobile app reported a significant decrease in patient activation. |
| Leichman et al. (2020)/ USA[16] | Customized Sleep Profile (CSP)/ Primary Study | 404 (P) | Caregivers of infants aged 6-11.9 months, recruitment over App profile entry | **Objective**: Examines the effectiveness of a custom sleep program in a mobile app using real-world data and assesses changes after following individualized recommendations for infants who were considered to be  problem sleepers (PS) by their caregivers versus those who were not considered to be problem sleepers (NPS). | **Design**: Questionnaire-based assessment of infant and child sleep by caregivers using an app for behavioral sleep intervention delivered via smartphone completed two time points: baseline and again 4 to 28 days later. **Primary parameter**: Caregivers completed the Brief Infant Sleep Questionnaire–Revised (BISQ-R) to rate infant and child sleep, based on sleep problem status (problem versus no problem sleepers). Changes in sleep patterns were analyzed in the app. | **Primary finding**: The BISQ-R Total score, total nighttime sleep, and total 24-hour sleep time improved for both groups, with a greater change for the PS group. Sleep onset latency improved in both groups. Earlier bedtimes, longer continuous stretches of sleep, as well as decreased number and duration of night wakings, were evident in the PS group only. |
| **Author (year)/ Country/ Reference** | **Study Project Name/ Intervention Type** | **Number of Participants (P)/ Apps (A)/ Studies (S)** | **Study population and recruitment/ included Apps/ included Studies** | **Objective** | **Methodology** | **Main Results** |
|  |  |  |  |  |  |  |
| De Lepeleere et al. (2017)/  Belgium[17] | Movie  Models/  Primary Study | 238 (P) | Parents of primary school children 6-12 years, recruited initially through schools, then social media (Facebook, EXPOO) | **Objective**: The effect of a health promoting online video intervention for parents (“Movie Models”) on children’s physical activity (PA), screen-time and healthy diet, and on specific parenting practices and parental self-efficacy related to these parenting practices was investigated. | **Design**: Two-armed, quasi experimental design for assessment of PA, screen-time, healthy diet and parenting practices. Intervention group participants were invited to watch online videos for 4 weeks then assessed at three time periods (baseline, one and at four months post baseline).  **Primary parameter**: Specific parenting practices, parental self-efficacy, PA, screen-time and healthy diet of the child were assessed at three time periods. Repeated Measures (Multivariate) ANOVAs were used to examine intervention effects. | **Primary finding**: Between T0 and T2, no significant intervention effects were found on children’s PA, screen-time or healthy diet. Most significant intervention effects were found for more complex parenting practices (e.g., an increase in motivating the child to eat fruit). Subgroup analyses showed that the intervention had more effect on the actual parenting practices related to PA, screen-time and healthy diet in parents of older children (10–12 years old), whereas intervention effects on parental self-efficacy related to those behaviors were stronger in parents of younger children (6–9 years old). |
| **Author (year)/ Country/ Reference** | **Study Project Name/ Intervention Type** | **Number of Participants (P)/ Apps (A)/ Studies (S)** | **Study population and recruitment/ included Apps/ included Studies** | **Objective** | **Methodology** | **Main Results** |
|  |  |  |  |  |  |  |
| Lozoya et al. (2019)/ USA[18] | Oral Health Smartphone Application/ Primary Study | 33 (P) | Parents of preschool children, recruited at preschools and via flyers | **Objective**: The purpose was to evaluate the effect of a smartphone app, based on the Theory of Planned Behavior (TPB), on the oral health behaviors of the parents of preschoolers. | **Design**: Two-phase, sequential, embedded mixed methods design exploring the app influence on attitudes, beliefs, perceived behavioral control, and intentions of parents of preschoolers. Phase 1: quasi-experimental, pretest-posttest design. Parents of preschool aged children participated in the 4-week intervention. Phase 2: qualitative interviews with a purposive sample of parents from Phase 1.  **Primary Parameter**: for Phase 1, a 124-item validated questionnaire measuring oral health behaviors in children and TPB determinants was used at two time points (before and after app use). Phase 2 consisted of semi-structured interviews using 13 open-ended questions. | **Primary finding**: Phase 1: Parents' behavioral intentions or oral health behaviors with their preschoolers did not significantly change from pre- to post-intervention (p>.05). Social norms (SN) and perceived behavioral control (PBC) predicted behavioral intentions pre-intervention and behavioral change post-intervention. Phase 2: Thematic analysis revealed that parents' belief in the importance of establishing oral health habits and brushing reminders and videos delivered via a mobile application supported efforts to form oral health habits. |
| **Author (year)/ Country/ Reference** | **Study Project Name/ Intervention Type** | **Number of Participants (P)/ Apps (A)/ Studies (S)** | **Study population and recruitment/ included Apps/ included Studies** | **Objective** | **Methodology** | **Main Results** |
|  |  |  |  |  |  |  |
| Mauch et al. (2021)/ Australia[19] | Review/  App Feasibility | 133 (P) | Working parent and main meal maker returning to work in the last 6 months, recruitment via Facebook, flyers and in childcare centers | **Objective**: Aims to determine the feasibility of existing commercially available apps for supporting the healthy food provision practices of working parents. | **Design**: Mixed-method app feasibility study for assessing selected meal planning and provision apps for families. Survey items were mapped to app features, with a subsample of parents (50,4%) allocated 2 apps each to trial simultaneously over 4 weeks. Afterwards, semi-structured interviews and a web-based survey app testing explored app utility and acceptability.  **Primary Parameter**: Baseline assessment of working parent meal provision needs was with a 10-item Capability, Opportunity, Motivation, and Behavior (COM-B) questionnaire. System Usability Scale, the user version of the Mobile App Rating Scale, and thematically analyzed interview data assessed feasibility and usability. | **Primary finding**: Participants identified a need for healthy recipes and time for food provision processes. Engagement quality was the lowest rated domain of the user version of the Mobile App Rating Scale across all 5 apps. The family organizer, requiring a high level of user input, was rated the lowest for usability. In the interviews, participants weighed the benefits of the apps (ie, time saving) against the effort involved in using them in determining their acceptability. Organization was a subtheme emerging from interviews, associated with the use of meal planners and shopping lists. Meal planners and shopping lists were used in time, while behavior was occurring. |
| **Author (year)/ Country/ Reference** | **Study Project Name/ Intervention Type** | **Number of Participants (P)/ Apps (A)/ Studies (S)** | **Study population and recruitment/ included Apps/ included Studies** | **Objective** | **Methodology** | **Main Results** |
|  |  |  |  |  |  |  |
| McKenzie et al. (2018)/ USA[20] | Make Safe Happen/  Study  Protocol | 1200 (P) | Parent or legal guardian of child 0-12 years, recruitment via random-digit-dial telephone calls, USPS mailings and advertising on social networking websites | **Objective**: Planned evaluation of the impact and effectiveness of a mobile app on safety and intended/actual behaviors for the prevention of unintentional home related injuries, compared with no intervention. | **Design**: Study protocol of a randomized controlled trial to evaluate a mobile technology-based safety behavior change intervention on parents’ safety knowledge and actions. Parents will complete a pretest survey, and will be randomized to receive the Make Safe Happen® app or a non-injury-related app, and then complete a posttest follow-up survey after 1 week. **Primary parameter**: safety knowledge; safety behaviors; safety device acquisition and use, and behavioral intention to take safety actions. | **Primary finding**: No results yet (study protocol) |
| **Author (year)/ Country/ Reference** | **Study Project Name/ Intervention Type** | **Number of Participants (P)/ Apps (A)/ Studies (S)** | **Study population and recruitment/ included Apps/ included Studies** | **Objective** | **Methodology** | **Main Results** |
|  |  |  |  |  |  |  |
| Meedya et al. (2020)/ Australia[21] | The Milky Way Program/ App Feasibility | 7 (P) | Pregnant women in the last trimester intending to breastfeed, recruitment via university news, flyers, & social media | **Objective**: Assesses the acceptability and usability of an app to support breastfeeding and finds care pathways with health care professionals. | **Design**: Mixed-method approach to pilot test the feasibily of a breastfeeding app at two time points. An online survey six weeks after birth and individual interviews at four months postpartum.  **Primary parameter**: The women’s feedback was collected through a co-design framework. | **Primary finding**: Women in the pilot study reported that the breastfeeding application was well designed, easy to use, interactive, reassuring and evidence-based with credible sources of information. |
| Meixner et al. (2019)/ Germany[22] | Gamification in health apps/  Survey Study | 1008 (P) | Persons of a German health insurance company, who have at least one child aged 8-16 years. | **Objective**: Health benefits of physical activity (PA) can be gained if the WHO recommendation for PA (≥30 minutes of moderate daily exercise and >150 minutes of exercise per week that makes the individual sweat or breathless) are fulfilled regularly. The aims of this study were to identify the PA and interests for digital components for health promotion, e.g. gamification, of key persons of the health promotion process within families in order to conduct targeted app interventions. | **Design**: Mixed-method design with a cross-sectional survey study and a preliminary qualitative survey to gain insights about the main contents of aspects that are expected within a health app.  **Primary parameter**: The assessment of efficiency and views on useful features and elements were also collected to provide a basis for concept development. The online questionnaire included the following sets of questions: socio-demography, health status and fields of action goals, physical activity, nutrition, relaxation, personality and motivation, smartphone usage, app feature and gamification. | **Primary finding**: 80.2 % of respondents do not fulfill WHO criteria for PA. Nevertheless, they want to increase their PA, e.g. with endurance and mobility interventions. Outdoor activities are also targeted, whereas institutional sports facilities seem unsuitable for these families. Interestingly, existing gamification elements in an app are of no interest to those respondents who do not meet the WHO criteria. |
| **Author (year)/ Country/ Reference** | **Study Project Name/ Intervention Type** | **Number of Participants (P)/**  **Apps (A)/ Studies (S)** | **Study population and recruitment/ included Apps/ included Studies** | **Objective** | **Methodology** | **Main Results** |
| Militello et al. (2021)/ USA[23] | Self-Management Intervention- Life Essentials (SMILE)/ App Feasibility | 19 (P) | Pregnant women with first child, recruited through a hospital newsletter and word of mouth. | **Objective**: to assess the feasibility of using voice technology to support perinatal health and infant care practices. | **Design**: Dental app feasibility study for pregnant women. Participants were asked to use the intervention, Self-Management Intervention–Life Essentials (SMILE), over the course of 2 weeks.  **Primary parameter**: SMILE provided users with perinatal health content delivered through mini podcasts (ranging from 3 to 8 minutes in duration). After each podcast, SMILE prompted users to provide immediate verbal feedback to the content. An exit interview was conducted with participants to gather feedback on the intervention. | **Primary finding**: Themes identified as important for perinatal health information include establishing routines, expected norms, and realistic expectations and providing key takeaways. Themes identified as important for voice interaction include customization and user preferences, privacy, family and friends, and context and convenience. Qualitative analysis suggested that perinatal health promotion content delivered by voice should be accurate and succinctly delivered and highlight key takeaways. Perinatal health interventions that use voice should provide users with the ability to customize the intervention but also provide opportunities to engage family members, particularly spouses. |
| **Author (year)/ Country/ Reference** | **Study Project Name/ Intervention Type** | **Number of Participants (P)/**  **Apps (A)/ Studies (S)** | **Study population and recruitment/ included Apps/ included Studies** | **Objective** | **Methodology** | **Main Results** |
|  |  |  |  |  |  |  |
| Otte et al. (2019)/ Netherlands[24] | uGrow app (medical-grade app dedicated to new parents for tracking their baby’s development)/  App Feasibility | 60 (P) | Chinese and American parents/ parents-to-be plus health care providers. Parents were recruited from a Web-based community website. | **Objective**: The aim of the study was twofold. First, it aimed to give a description of the development process of the insights for the uGrow app. Second, it aimed to present results from a study about parents’ experiences with the insights. | **Design**: Mixed method study. The development process comprised 3 phases: a formative phase, development phase, and summative phase. In the formative phase, 3 sub-studies were executed in series to understand and identify parents’ and health care professionals’ (HCPs) needs for insights, using qualitative and quantitative methods.  **Primary parameter**: After the formative phase, insights were created during the development phase. Subsequently, in the summative phase, these insights were validated against parents’ experience using a quantitative approach. | **Primary finding**: Parents indicated having a need for smart information based on a data analysis of the data they track in an app. HCPs supported the general concept of insights for the uGrow app, although specific types of insights were considered irrelevant or even risky. After implementing a preliminary set of insights in a prototype version of the uGrow app and testing it with parents, the majority of parents (87%) reported being satisfied with the insights. A total of 89 insights were implemented in a final version of the uGrow app. In the summative phase, the majority of parents reported experiencing these insights as reassuring and useful (94%), as adding enjoyment (85%), and as motivating for continuing tracking for a longer period of time (77%). |
| **Author (year)/ Country/ Reference** | **Study Project Name/ Intervention Type** | **Number of Participants (P)/**  **Apps (A)/ Studies (S)** | **Study population and recruitment/ included Apps/ included Studies** | **Objective** | **Methodology** | **Main Results** |
|  |  |  |  |  |  |  |
| Pond et al. (2019)/ Australia[25] | SWAP IT Childcare trial/  Study protocol | Approximately  390 (P) | Parents from 18 centre-based childcare services in New South Wales/Australia. | **Objective**: The study aims to assess the impact of a mobile health (m-health) intervention on reducing the packing of discretionary foods in children’s childcare lunchboxes. | **Design**: Planned cluster randomized controlled trial design. Randomized services to receive either a 4-month m-health intervention (SWAP IT Childcare) or usual care. Intervention development informed by the Behavior Change Wheel model. Information provision for parents will target lunchbox food with guidelines and website links. Push notifications planned via an existing app used by childcare services to communicate with parents and carers.  **Primary parameter**: Lunch box foods assessed by measured: energy (kilojoules) from discretionary, total energy (kilojoules), saturated fat (grams), total and added sugars (grams) and sodium (milligrams) from all foods packed in lunchboxes. Outcomes will be assessed by weighing and photographing all lunchbox food items at baseline and at the end of the intervention. | **Primary finding**: No results yet (study protocol). |
| **Author (year)/ Country/ Reference** | **Study Project Name/ Intervention Type** | **Number of Participants (P)/**  **Apps (A)/ Studies (S)** | **Study population and recruitment/ included Apps/ included Studies** | **Objective** | **Methodology** | **Main Results** |
|  |  |  |  |  |  |  |
| Roberts et al. (2019)/ USA[26] | Make Safe Happen/ App Feasibility | 49 (P) | Parents of children 0-12 years, recruited through a market research firm | **Objective**: to evaluate parental injury prevention awareness and home safety behaviors, motivations for and challenges to taking injury prevention and safety actions for parents as well as user experience following the use of the Make Safe Happen mobile app. | **Design**: Focus group-based app feasibility study followed by a survey component: (1) a pretest survey, (2) use of the Make Safe Happen app, and (3) a posttest survey.  **Primary parameter**: focus groups were analyzed via thematic analysis. Pretest and posttest difference of the mean total safety  knowledge score was analyzed with the chi-square test using alpha=.05. | **Primary finding**: Following the use of the Make Safe Happen app, parents reported a significant increase in injury prevention awareness and completed 45% more home safety behaviors in and around their homes. Nearly all of the parents felt the app provided them with the information needed to make their home safer for their children; the great majority of parents intended to make such changes in the future. |
| **Author (year)/ Country/ Reference** | **Study Project Name/ Intervention Type** | **Number of Participants (P)/**  **Apps (A)/ Studies (S)** | **Study population and recruitment/ included Apps/ included Studies** | **Objective** | **Methodology** | **Main Results** |
|  |  |  |  |  |  |  |
| Roed et al. (2021)/  Norway[27] | Food4 toddlers/ Primary Study | 404 (P) | Parents of infants and toddlers, recruited through tailored social media advertisement | **Objective**: The effect of the Food4toddlers eHealth intervention was assessed, which aimed to enhance toddlers' diets by shaping their food and eating environment. | **Design**: Randomized controlled trial with parents that completed an online questionnaire at three time periods (baseline, post-intervention, and 6 months after the intervention). The intervention was guided by social cognitive theory. The intervention group used the Food4toddlers website for 6 months.  **Primary parameter**: Intervention effects were measured on child diet from baseline to follow-up 1 and from baseline to follow-up 2, Between- group differences in changes over time for frequency and variety of fruits and vegetables and frequency of discretionary foods were assessed. | **Primary finding**: At follow-up 1, a significant time × group interaction was observed for the frequency of vegetable intake (P=.02). The difference between groups in the change from baseline to follow-up 1 was 0.46 vegetable items per day (95% CI 0.06-0.86) in favor of the intervention group. No other significant between-group differences in dietary changes from baseline to follow-up 1 or follow-up 2 were observed. However, there is a clear time trend showing that the intake of discretionary foods increases by time from less than 1 item per week at baseline to more than 4 items per week at 2 years of age (P<.001), regardless of group. |
| **Author (year)/ Country/ Reference** | **Study Project Name/ Intervention Type** | **Number of Participants (P)/**  **Apps (A)/ Studies (S)** | **Study population and recruitment/ included Apps/ included Studies** | **Objective** | **Methodology** | **Main Results** |
|  |  |  |  |  |  |  |
| Russel et al. (2018)/ Australia[28] | The Growing Healthy Program/ Primary Study | 645 (P) | Pregnant women (30+ weeks gestation) or parents of infant under 3 months, recruited via web advertising | **Objective**: to describe the effects of an mHealth intervention on parental feeding practices, infant food preferences, and infant satiety responsiveness. | **Design**: Quasi-experimental study that was conducted with an app (Growing Healthy) and a nonrandomized comparison group (“Baby's First Food"). The intervention group received access to a free app with push notifications, a website, and an online forum that provided them with evidence-based advice on infant feeding for heathy growth from birth until 9 month of age. Behavior change techniques were selected using the Behavior Change Wheel framework. Participants in both groups completed three web-based surveys at three time points (at 3, 6 and 9 months).  **Primary parameter**: Surveys included questions on infant feeding practices and beliefs (Infant Feeding Questionnaire, IFQ), satiety responsiveness (Baby Eating Behavior Questionnaire), and infant’s food exposure and liking. Multivariate linear regression models were adjusted and then multivariate logistic regression was performed. | **Primary finding**: All but one (IFQ subscale “concerns about infant overeating or becoming overweight” at T2) of the measured outcomes did not differ between Growing Healthy and Baby's First Food. |
| **Author (year)/ Country/ Reference** | **Study Project Name/ Intervention Type** | **Number of Participants (P)/**  **Apps (A)/ Studies (S)** | **Study population and recruitment/ included Apps/ included Studies** | **Objective** | **Methodology** | **Main Results** |
|  |  |  |  |  |  |  |
| Scott et al. (2021)/ Australia[29] | The Parent Infant Feeding Initiative (PIFI)/ Primary Study | 1426 (P) | Expecting couples, recruited from antenatal classes at hospitals. | **Objective**: to determine the effectiveness of various father-focused breastfeeding interventions in terms of key infant feeding outcomes. | **Design**: a 4-arm, factorial, randomized controlled trial. The trial arms included a control group and 3 interventions, consisting of a face-to-face father-focused antenatal breastfeeding class facilitated by a male peer facilitator; Milk Man, a breastfeeding smartphone app designed specifically for fathers; and a combination of both interventions. Expecting couples randomized to 1 of the 4 arms. Each partner completed surveys at recruitment and at 6 weeks and 26 weeks postpartum.  **Primary parameter**: Primary outcomes were duration of exclusive and any breastfeeding. Secondary outcomes included age of introduction of formula and complementary foods, maternal breastfeeding self-efficacy, and partner postpartum support. | **Primary finding**: 76.6% of fathers completed the baseline questionnaire, 58.6% completed the 6-week follow-up questionnaire, and 49.2% completed the 26-week follow-up questionnaire. There were no significant differences between the control and any of the intervention groups in any of the infant feeding outcomes or level of breastfeeding self-efficacy and postpartum partner support reported by mothers. |
| **Author (year)/ Country/ Reference** | **Study Project Name/ Intervention Type** | **Number of Participants (P)/**  **Apps (A)/ Studies (S)** | **Study population and recruitment/ included Apps/ included Studies** | **Objective** | **Methodology** | **Main Results** |
|  |  |  |  |  |  |  |
| Shorey et al. (2019)/ Singapore[30] | Supportive educational parenting program (SEPP)/ Primary Study | 236 (P) | 118 heterosexual married couple dyads, recruited at antenatal checkup appointments at antenatal clinics | **Objective**: to examine the effectiveness of a technology-based supportive educational parenting program (SEPP) on parenting outcomes during the perinatal period in couples. | **Design**: Randomized, single-blinded, parallel-armed, controlled trial. New parents were randomly assigned to the intervention or control group. The theory-based Intervention for parenting after birth included: 2 telephone-based educational sessions (1 antenatal and 1 immediately postnatal) and 1 month mobile health app follow-up. Control group received routine perinatal care. Data were collected over 6 months at 4 time points (third trimester of pregnancy, and 2 days, 1 month, and 3 months postpartum.  **Primary parameter**: measures including parenting self-efficacy (PSE), parental bonding, perceived social support, parenting satisfaction, postnatal depression (PND), and anxiety were measured using reliable and valid instruments. | **Primary finding**: The intervention group showed significantly better outcome scores than the control group from baseline to 3 months postpartum for PSE (mean difference, MD, 0.37; 95% CI 0.06 to 0.68; P=.02), parental bonding (MD −1.32; 95% CI −1.89 to −0.75; P<.001), self-perceived social support (MD 0.69; 95% CI 0.18 to 1.19; P=.01), parenting satisfaction (MD 1.40; 95% CI 0.86 to 1.93; P<.001), and PND (MD −0.91; 95% CI −1.34 to −0.49; P<.001). Postnatal anxiety (PNA) scores of the intervention group were only significantly better after adjusting for covariates (MD −0.82; 95% CI −1.15 to −0.49; P<.001). |
| **Author (year)/ Country/ Reference** | **Study Project Name/ Intervention Type** | **Number of Participants (P)/**  **Apps (A)/ Studies (S)** | **Study population and recruitment/ included Apps/ included Studies** | **Objective** | **Methodology** | **Main Results** |
| Sun et al. (2017)/ USA[31] | 5-4-3-2-1-0-Go! Pro-gram/  Primary Study | 32 (P) | Mother-child  dyads with self-identifying Chinese mothers,  recruited through a Head Start Program. | **Objective**: Assesses the feasibility and impact of a family-centered, technology-based intervention to improve the health behaviors of low-income, overweight, or obese Chinese mothers and their children, guided by the Information Motivation Behavioral Skills Model. | **Design**: The randomized pilot study consisted of 8 weekly sessions, examined feasibility of a tablet computer–based intervention among 32 mother–child dyads. The study estimated effect size of the intervention at 3 time periods (baseline, 3 and 6 months)  **Primary parameter**: maternal outcomes including self-efficacy, eating behaviors, physical activity, child-feeding practices, and change in body mass index. | **Primary finding**: The tablet computer–based intervention is feasible among low-income Chinese mothers with low acculturation. A large-effect size was observed in reducing maternal body mass index, waist circumference, and improving maternal eating style and self-efficacy for promoting healthy eating. |
| **Author (year)/ Country/ Reference** | **Study Project Name/ Intervention Type** | **Number of Participants (P)/**  **Apps (A)/ Studies (S)** | **Study population and recruitment/ included Apps/ included Studies** | **Objective** | **Methodology** | **Main Results** |
| Sutherland et al. (2019)/ Australia[32] | SWAP IT/ Primary Study | 1119 (P) | Families (parents, children, lunchboxes & schools), recruited from schools. | **Objective**: assessed the potential efficacy, feasibility and acceptability of an m-health intervention, ‘SWAP IT’, to improve the energy and nutritional quality of foods packed in children’s lunchboxes. | **Design**: a 2X2 factorial cluster randomized-controlled trial design assessing the effects of the lunchbox intervention only. The lunchbox intervention comprised four strategies: 1) school nutrition guidelines; 2) lunchbox lessons; 3) information pushed to parents via a school-communication app and 4) parent resources addressing barriers to packing healthy lunchboxes.  **Primary parameter**: Outcome measures were taken at baseline and immediately post-intervention (10 weeks) and included measures of effectiveness (mean energy (kJ) packed in lunchboxes, total energy and percentage energy from recommended foods, feasibility and acceptability to school staff and parents. Linear mixed models were used to assess intervention efficacy. | **Primary finding**: Of the 1915 lunchbox observations, at follow-up there was no significant differences between intervention and control group in mean energy of foods packed within lunchboxes. There was a significant increase favouring the intervention in the secondary outcome of mean lunchbox energy from recommended foods (79.21 kJ, CI = 1.99, 156.43, p = 0.04), and a non-significant increase in percentage of lunchbox energy from recommended foods in intervention schools (4.57%, CI = -0.52, 9.66, p = 0.08). The views of the messages pushed via the app ranged from 387 to 1550 views per week. A large proportion (71%) of parents reported awareness of the intervention, making healthier swaps in the lunchbox (55%), and pushed content was helpful (84%). |
| **Author (year)/ Country/ Reference** | **Study Project Name/ Intervention Type** | **Number of Participants (P)/**  **Apps (A)/ Studies (S)** | **Study population and recruitment/ included Apps/ included Studies** | **Objective** | **Methodology** | **Main Results** |
| Taki et al. (2019)/ Australia[33] | The Growing Healthy Program/ Primary Study | 21 (P) | Pregnant women (30+ weeks gestation) or parents of infant under 3 months, recruited via web advertising, face-to-face in parenting groups, through practitioners. | **Objective**: The Growing healthy (GH) program is the first obesity prevention program delivered via a smartphone app and website offering evidence-based information on infant feeding from birth until 9 months of age. This sub-study aimed to explore how the design features, quality of the app and participant characteristics influenced parents' engagement with the GH app. | **Design**: sequential mixed methods design was used to evaluate the use by parent participants of an infant feeding app.  **Primary parameter**: Participant app engagement was measured through a purpose-built Engagement Index (EI) using app metrics. Participants were categorized as low, moderately or highly engaged based on their EI score upon completing the 9 months program and were then invited to participate in semi-structured telephone interviews exploring factors that influenced app engagement including delivery features and quality. Thematic analysis networks was used for analysis. | **Primary finding**: 108/225 expressed interest and 18 interviews were conducted from low (n = 3), moderately (n = 7), or highly (n = 8) engaged participants based on purposeful sampling. Participants defined as highly engaged were likely to be a first-time parent, felt the app content to be trustworthy and the app design facilitated easy navigation and regularly opened the push notifications. Participants defined as having low or moderate engagement were likely to have experience from previous children, felt they had sufficient knowledge on infant feeding and the app did not provide further information, or experienced technological issues including app dysfunction due to system upgrades. |
| Trost & Brookes (2021)/ Australia[34] | Moovosity/ Primary Study | 34 (P) | Parent-child dyads, recruited through a university email list service for academic and professional staff and by word of mouth. | **Objective**: Evaluates the effectiveness of a digital application to increase fundamental movement skill proficiency, physical activity levels in preschool-aged children and examines parental support. | **Design**: A randomized controlled trial (RCT) was conducted with parent-child dyads randomly assigned to either the 8-week intervention or wait-list control condition. Fundamental movement skills proficiency, physical activity (PA), and parental support for PA was assessed at baseline and 8-weeks.  **Primary parameter**:  Group differences for pre to post changes in outcomes were tested for significance using general linear mixed models. | **Primary finding**: There was a significant group by time interaction for object control skills (F_1,32_ = 10.81, *P* = 0.003). Intervention children exhibited significant improvements in object control skills, while children in the wait-list control group exhibited a modest decline. Intervention children also exhibited improvements in locomotor skills, while wait-listed controls exhibited minimal change; however, the group by time interaction fell outside the 0.05 level of significance (F_1,32_ = 3.15, *P* = 0.09). There were no significant intervention effects observed for child PA (F_1,32_ = 0.03, *P* = 0.86) and parental support for PA (F_1,32_ = 0.84, *P* = 0.37). |
| **Author (year)/ Country/ Reference** | **Study Project Name/ Intervention Type** | **Number of Participants (P)/**  **Apps (A)/ Studies (S)** | **Study population and recruitment/ included Apps/ included Studies** | **Objective** | **Methodology** | **Main Results** |
|  |  |  |  |  |  |  |
| Venezia et al. (2019)/ USA[35] | Let’s Wiggle with 5-2-1-0/  App Feasibility | 7 (P) | Parents (all mothers), recruited from a childcare center | **Objective**: Assesses the effectiveness of educational tools and a physical activity app for parents of preschool children to use in home-based settings. | **Design**: App feasibility was assessed through interviews with parents/guardians of pre-school age children on the desired content of a physical activity app targeted at children.  **Primary parameter**: Following a review of the free physical activity app, app content, imagery, resources, and overall user friendliness were analyzed using thematic analysis. | **Primary finding**: Parents suggested that the app be frequently updated with new videos and that more colorful icons that better represent the physical activity videos be added. In addition, they desired access to printable resources such as curriculum cards and coloring pages. Participants identified that the app was easy to follow and they would recommend the app to a friend. The majority of recommendations included improving the presentation of the app so that it looks more appealing and needing the app to be more preschool-age friendly. |
| **Author (year)/ Country/ Reference** | **Study Project Name/ Intervention Type** | **Number of Participants (P)/**  **Apps (A)/ Studies (S)** | **Study population and recruitment/ included Apps/ included Studies** | **Objective** | **Methodology** | **Main Results** |
|  |  |  |  |  |  |  |
| Winders et al. (2017)/ USA[36] | Literature/ App Review | 46 (A) | 46 Apps, relevant to parenting, child or infant health, found on iTunes App Store | **Objective**: A review of available apps for new parents with an evaluation of the information and functionality. | **Design**: A review of parent apps assessing relevant parenting, child health, or infant health content, in English, containing parent educationcontent.  **Primary parameter**: The Patient Education Materials Assessment Tool was used to evaluate the health literacy subscales called Understandability and Actionability. Content analysis included Authority, Objectivity, Accuracy, Timeliness, and Usability. | **Primary finding**: The majority of the apps (70%) were in English only. The price ranged from free to $4.99. The purpose, target audience, and topics varied. Although all included apps were for parents, some were for more targeted groups of parents. The source of the information was not presented in 26% of the apps. Most apps took the user to a Web site or an article to read. Functionality of the apps was limited, with none of them providing a customized experience. |
| Wunsch et al. (2020)/ Germany[37] | Smartfamily (SF2.0 trial)/ Stud Protocol | 156 (P) | Families, at least one parent and a child aged 10 or older. Participants will be recruited in schools, sports clubs and via personal and public communication. | **Objective**: Develops, refines, and evaluates an mHealth intervention to improve physical activity levels and healthy eating at the individual and family level. | **Design**: Planned cluster randomized controlled trial of a smartphone app based on behavior change theories and behavior change techniques to be evaluated with family members individually and cooperatively with 3 measurement periods. The intervention group uses the app for 3 consecutive weeks, whereas the control group receives no treatment.  **Primary parameter**: Physical activity and healthy eating Secondary Outcomes: intrinsic motivation, behavior-specific self-efficacy, and the family health climate, complemented by an intentional measure. Trial results will be used to re-evaluate the app. | **Primary finding**: No results yet (study protocol). |
| **Author (year)/ Country/ Reference** | **Study Project Name/ Intervention Type** | **Number of Participants (P)/**  **Apps (A)/ Studies (S)** | **Study population and recruitment/ included Apps/ included Studies** | **Objective** | **Methodology** | **Main Results** |
| Zarnowiecki et al. (2020)/ Australia[38] | Literature/ App Review | 11 (S) | 11 Studies, Digital interventions (websites or apps) targeting parents or families were included where the nutrition component was at least 50% of content. | **Objective**: to conduct a comprehensive and systematic evaluation of nutrition promotion via websites and apps supporting parents to influence children’s nutrition, from three different perspectives | **Design**: Three systematic reviews were undertaken children’s nutrition, from three different perspectives: 1) current evidence base, 2) end user (parent) experience and 3) current commercial offerings.  **Primary parameter**: Search results were extracted into standardized spreadsheets and quality appraisal of included search results assessed through the The Mobile App Rating Scale (MARS). | **Primary finding**: Studies evaluating digital nutrition interventions targeting parents (*n* = 11) demonstrated effectiveness for improving nutrition outcomes, self-efficacy and knowledge. Six of the included randomized controlled trials reported digital interventions to be equal to, or better than comparison groups. User-testing studies (*n* = 9) identified that digital platforms should include both informative content and interactive features. Parents wanted evidence-based information from credible sources, practical tools, engaging content and connection with other users and health professionals. Websites targeting lunch provision (*n* = 15) were developed primarily by credible sources and included information-based content consistent with dietary guidelines and limited interactive features. Lunchbox apps (*n* = 6), developed mostly by commercial organisations, were more interactive but provided less credible information. |
| **Author (year)/ Country/ Reference** | Study Project Name/ Intervention Type | **Number of Participants (P)/**  **Apps (A)/ Studies (S)** | **Study population and recruitment/ included Apps/ included Studies** | **Objective** | **Methodology** | **Main Results** |
| Zolfaghari et al. (2021)/ Iran[39] | Gamified smart phone mobile health application for oral health promotion/ Primary Study | 58 (P) | Mother-child pairs, mothers of preschool children recruited while attending specialty dental clinic. | **Objective**: Tests the design of a mobile app and assesses the effect of gamification on the oral health of children by mothers. | **Design**: A randomized controlled clinical trial with a pretest and posttest evaluation of oral health knowledge and practice of mothers before and after an intervention of using a simple vs. gamified app containing information about early childhood caries, health diet, sugars, baby-oral hygiene, fluoride effect, fluoride toothpaste, tooth- brushing training video and regular dental visits.  **Primary parameter**: evaluated questionnaire regarding oral health knowledge. | **Primary finding**: The mean knowledge score of mothers in the pretest was 10.5 and 11.3 in simple app and gamified app group, respectively, which changed to 13.1 and 14.3, respectively in the posttest. The mean practice score of mothers was 4.4 and 4.8 in simple app and gamified app groups, respectively in the pretest, which changed to 8.5 and 8, respectively in the posttest. The mean dental plaque index of children in the pretest was 0.8 and 1 in simple app and gamified app groups, respectively, which changed to 0.5 and 0.5, respectively in the posttest. Children had better Plaque control in gamified app group (*P* < 0.05). |
|  |  |  |  |  |  |  |

**Literature:**

1. Au LE, Whaley S, Rosen NJ, Meza M, Ritchie LD. Online and In-Person Nutrition Education Improves Breakfast Knowledge, Attitudes, and Behaviors: A Randomized Trial of Participants in the Special Supplemental Nutrition Program for Women, Infants, and Children. J Acad Nutr Diet. 2016 Mar;116(3):490-500. PMID: 26669795. doi: 10.1016/j.jand.2015.10.012.

2. Biviji R, Vest JR, Dixon BE, Cullen T, Harle CA. Content analysis of behavior change techniques in maternal and infant health apps. Translational Behavioral Medicine. 2021;11(2):504-15. doi: 10.1093/tbm/ibaa039.

3. Burgess J, Watt K, Kimble RM, Cameron CM. Combining Technology and Research to Prevent Scald Injuries (the Cool Runnings Intervention): Randomized Controlled Trial. J Med Internet Res. 2018 Oct 10;20(10):e10361. PMID: 30305263. doi: 10.2196/10361.

4. Cheng H, Tutt A, Llewellyn C, Size D, Jones J, Taki S, et al. Content and Quality of Infant Feeding Smartphone Apps: Five-Year Update on a Systematic Search and Evaluation. JMIR Mhealth Uhealth. 2020 May 27;8(5):e17300. PMID: 32459187. doi: 10.2196/17300.

5. Choi J, Lee JH, Vittinghoff E, Fukuoka Y. mHealth Physical Activity Intervention: A Randomized Pilot Study in Physically Inactive Pregnant Women. Matern Child Health J. 2016 May;20(5):1091-101. PMID: 26649879. doi: 10.1007/s10995-015-1895-7.

6. Cullen KW, Thompson D, Chen TA. Outcome Evaluation of Family Eats. Health Educ Behav. 2017 Feb;44(1):32-40. PMID: 27198535. doi: 10.1177/1090198116643917.

7. Cushing CC, Fedele DA, Brannon EE, Kichline T. Parents' Perspectives on the Theoretical Domains Framework Elements Needed in a Pediatric Health Behavior App: A Crowdsourced Social Validity Study. Jmir Mhealth and Uhealth. 2018 Dec;6(12). PMID: WOS:000454340000001. doi: 10.2196/mhealth.9808.

8. Deave T, Ginja S, Goodenough T, Bailey E, Piwek L, Coad J, et al. The Bumps and BaBies Longitudinal Study (BaBBLeS): a multi-site cohort study of first-time mothers to evaluate the effectiveness of the Baby Buddy app. Mhealth. 2019 2019/9/25;5:42. doi: 10.21037/mhealth.2019.08.05.

9. Dodd JM, Louise J, Cramp C, Grivell RM, Moran LJ, Deussen AR. Evaluation of a smartphone nutrition and physical activity application to provide lifestyle advice to pregnant women: The SNAPP randomised trial. Matern Child Nutr. 2018 Jan;14(1). PMID: 28836373. doi: 10.1111/mcn.12502.

10. Gomes AI, Pereira AI, Roberto MS, Boraska K, Barros L. Changing parental feeding practices through web-based interventions: A systematic review and meta-analysis. PLoS One. 2021;16(4):e0250231. PMID: 33909666. doi: 10.1371/journal.pone.0250231.

11. Helle C, Hillesund ER, Omholt ML, Øverby NC. Early food for future health: a randomized controlled trial evaluating the effect of an eHealth intervention aiming to promote healthy food habits from early childhood. BMC Public Health. 2017 2017/9/20;17(1):729. doi: 10.1186/s12889-017-4731-8.

12. Henriksson H, Alexandrou C, Henriksson P, Henström M, Bendtsen M, Thomas K, et al. MINISTOP 2.0: a smartphone app integrated in primary child health care to promote healthy diet and physical activity behaviours and prevent obesity in preschool-aged children: protocol for a hybrid design effectiveness-implementation study. BMC Public Health. 2020 Nov 23;20(1):1756. PMID: 33228572. doi: 10.1186/s12889-020-09808-w.

13. Knowlden A, Sharma M. One-Year Efficacy Testing of Enabling Mothers to Prevent Pediatric Obesity Through Web-Based Education and Reciprocal Determinism (EMPOWER) Randomized Control Trial. Health Educ Behav. 2016 Feb;43(1):94-106. PMID: 26272782. doi: 10.1177/1090198115596737.

14. Laws RA, Denney-Wilson EA, Taki S, Russell CG, Zheng M, Litterbach EK, et al. Key Lessons and Impact of the Growing Healthy mHealth Program on Milk Feeding, Timing of Introduction of Solids, and Infant Growth: Quasi-Experimental Study. JMIR Mhealth Uhealth. 2018 Apr 19;6(4):e78. PMID: 29674313. doi: 10.2196/mhealth.9040.

15. Ledford CJW, Womack JJ, Rider HA, Seehusen AB, Conner SJ, Lauters RA, et al. Unexpected Effects of a System-Distributed Mobile Application in Maternity Care: A Randomized Controlled Trial. Health Educ Behav. 2018 Jun;45(3):323-30. PMID: 28918669. doi: 10.1177/1090198117732110.

16. Leichman ES, Gould RA, Williamson AA, Walters RM, Mindell JA. Effectiveness of an mHealth Intervention for Infant Sleep Disturbances. Behav Ther. 2020 Jul;51(4):548-58. PMID: 32586429. doi: 10.1016/j.beth.2019.12.011.

17. De Lepeleere S, De Bourdeaudhuij I, Cardon G, Verloigne M. The effect of an online video intervention 'Movie Models' on specific parenting practices and parental self-efficacy related to children's physical activity, screen-time and healthy diet: a quasi experimental study. BMC Public Health. 2017 Apr 27;17(1):366. PMID: 28449658. doi: 10.1186/s12889-017-4264-1.

18. Lozoya CJS, Giblin-Scanlon L, Boyd LD, Nolen S, Vineyard J. Influence of a Smartphone Application on the Oral Health Practices and Behaviors of Parents of Preschool Children. J Dent Hyg. 2019 Oct;93(5):6-14. PMID: 31628171.

19. Mauch CE, Laws RA, Prichard I, Maeder AJ, Wycherley TP, Golley RK. Commercially Available Apps to Support Healthy Family Meals: User Testing of App Utility, Acceptability, and Engagement. JMIR Mhealth Uhealth. 2021 May 7;9(5):e22990. PMID: 33960951. doi: 10.2196/22990.

20. McKenzie LB, Roberts KJ, Clark R, McAdams R, Abdel-Rasoul M, Klein EG, et al. A randomized controlled trial to evaluate the Make Safe Happen® app-a mobile technology-based safety behavior change intervention for increasing parents' safety knowledge and actions. Inj Epidemiol. 2018 Mar 12;5(1):5. PMID: 29527644. doi: 10.1186/s40621-018-0133-3.

21. Meedya S, Win K, Yeatman H, Fahy K, Walton K, Burgess L, et al. Developing and testing a mobile application for breastfeeding support: the milky way application. Women and Birth. 2021;34(2):e196-e203. doi: <https://doi.org/10.1016/j.wombi.2020.02.006>.

22. Meixner C, Baumann H, Fenger A, Wollesen B, editors. Gamification in health apps to increase physical activity within families. 2019 International Conference on Wireless and Mobile Computing, Networking and Communications (WiMob); 2019 21-23 Oct. 2019.

23. Militello L, Sezgin E, Huang Y, Lin S. Delivering Perinatal Health Information via a Voice Interactive App (SMILE): Mixed Methods Feasibility Study. JMIR Form Res. 2021 Mar 1;5(3):e18240. PMID: 33646136. doi: 10.2196/18240.

24. Otte RA, van Beukering AJE, Boelens-Brockhuis LM. Tracker-Based Personal Advice to Support the Baby's Healthy Development in a Novel Parenting App: Data-Driven Innovation. JMIR Mhealth Uhealth. 2019 Jul 24;7(7):e12666. PMID: 31342901. doi: 10.2196/12666.

25. Pond N, Finch M, Sutherland R, Wolfenden L, Nathan N, Kingsland M, et al. Cluster randomised controlled trial of an m-health intervention in centre-based childcare services to reduce the packing of discretionary foods in children's lunchboxes: study protocol for the 'SWAP IT Childcare' trial. BMJ Open. 2019 Jun 1;9(5):e026829. PMID: 31154306. doi: 10.1136/bmjopen-2018-026829.

26. Roberts KJ, McAdams RJ, Kristel OV, Szymanski AM, McKenzie LB. Qualitative and Quantitative Evaluation of the Make Safe Happen App: Mobile Technology-Based Safety Behavior Change Intervention for Parents. JMIR Pediatr Parent. 2019 Mar 14;2(1):e12022. PMID: 31518322. doi: 10.2196/12022.

27. Røed M, Medin AC, Vik FN, Hillesund ER, Van Lippevelde W, Campbell K, et al. Effect of a Parent-Focused eHealth Intervention on Children's Fruit, Vegetable, and Discretionary Food Intake (Food4toddlers): Randomized Controlled Trial. J Med Internet Res. 2021 Feb 16;23(2):e18311. PMID: 33591279. doi: 10.2196/18311.

28. Russell CG, Denney-Wilson E, Laws RA, Abbott G, Zheng MB, Lymer SJ, et al. Impact of the Growing Healthy mHealth Program on Maternal Feeding Practices, Infant Food Preferences, and Satiety Responsiveness: Quasi-Experimental Study. Jmir Mhealth and Uhealth. 2018 Apr;6(4). PMID: WOS:000488249000005. doi: 10.2196/mhealth.9303.

29. Scott JA, Burns SK, Hauck YL, Giglia RC, Jorgensen AM, White BK, et al. Impact of a Face-To-Face Versus Smartphone App Versus Combined Breastfeeding Intervention Targeting Fathers: Randomized Controlled Trial. JMIR Pediatr Parent. 2021 Apr 12;4(2):e24579. PMID: 33843604. doi: 10.2196/24579.

30. Shorey S, Ng YPM, Ng ED, Siew AL, Mörelius E, Yoong J, et al. Effectiveness of a Technology-Based Supportive Educational Parenting Program on Parental Outcomes (Part 1): randomized Controlled Trial. Journal of medical Internet research. 2019;21(2):e10816. PMID: CN-01792502. doi: 10.2196/10816.

31. Sun A, Cheng J, Bui Q, Liang Y, Ng T, Chen JL. Home-Based and Technology-Centered Childhood Obesity Prevention for Chinese Mothers With Preschool-Aged Children. J Transcult Nurs. 2017 Nov;28(6):616-24. PMID: 28826348. doi: 10.1177/1043659617719139.

32. Sutherland R, Nathan N, Brown A, Yoong S, Finch M, Lecathelinais C, et al. A randomized controlled trial to assess the potential efficacy, feasibility and acceptability of an m-health intervention targeting parents of school aged children to improve the nutritional quality of foods packed in the lunchbox 'SWAP IT'. Int J Behav Nutr Phys Act. 2019 Jul 2;16(1):54. PMID: 31266506. doi: 10.1186/s12966-019-0812-7.

33. Taki S, Russell CG, Lymer S, Laws R, Campbell K, Appleton J, et al. A Mixed Methods Study to Explore the Effects of Program Design Elements and Participant Characteristics on Parents' Engagement With an mHealth Program to Promote Healthy Infant Feeding: The Growing Healthy Program. Front Endocrinol (Lausanne). 2019;10:397. PMID: 31293515. doi: 10.3389/fendo.2019.00397.

34. Trost SG, Brookes DS. Effectiveness of a novel digital application to promote fundamental movement skills in 3-to 6-year-old children: A randomized controlled trial. Journal of Sports Sciences. 2021;39(4):453-9.

35. Venezia AP, Wirth CK, Vinci DM. Active Families at Home: The Development of a Let's Wiggle With 5-2-1-0 App. Journal Articles

Reports - Research. 2019.

36. Winders D, Logsdon MC, Vogt K, Rushton J, Myers J, Lauf A, et al. Parent EDUCATION is CHANGING: A REVIEW OF SMARTPHONE APPS. Mcn-the American Journal of Maternal-Child Nursing. 2017 Sep-Oct;42(5):248-56. PMID: WOS:000408521000002. doi: 10.1097/nmc.0000000000000353.

37. Wunsch K, Eckert T, Fiedler J, Cleven L, Niermann C, Reiterer H, et al. Effects of a Collective Family-Based Mobile Health Intervention Called "SMARTFAMILY" on Promoting Physical Activity and Healthy Eating: Protocol for a Randomized Controlled Trial. JMIR Res Protoc. 2020 2020/11/11;9(11):e20534. doi: 10.2196/20534.

38. Zarnowiecki D, Mauch CE, Middleton G, Matwiejczyk L, Watson WL, Dibbs J, et al. A systematic evaluation of digital nutrition promotion websites and apps for supporting parents to influence children's nutrition. Int J Behav Nutr Phys Act. 2020 2020/2/10;17(1):17. doi: 10.1186/s12966-020-0915-1.

39. Zolfaghari M, Shirmohammadi M, Shahhosseini H, Mokhtaran M, Mohebbi SZ. Development and evaluation of a gamified smart phone mobile health application for oral health promotion in early childhood: a randomized controlled trial. BMC Oral Health. 2021 Jan 7;21(1):18. PMID: 33413304. doi: 10.1186/s12903-020-01374-2.
