# Supplementary figures and images for: Planting Seeds for the Future: Scoping Review of Child Health Promotion Apps for Parents
Source: JMIR Mhealth Uhealth. 2023 Jul 20;11:e39929. doi: 10.2196/39929 (PMC10401193; doi:10.2196/39929)

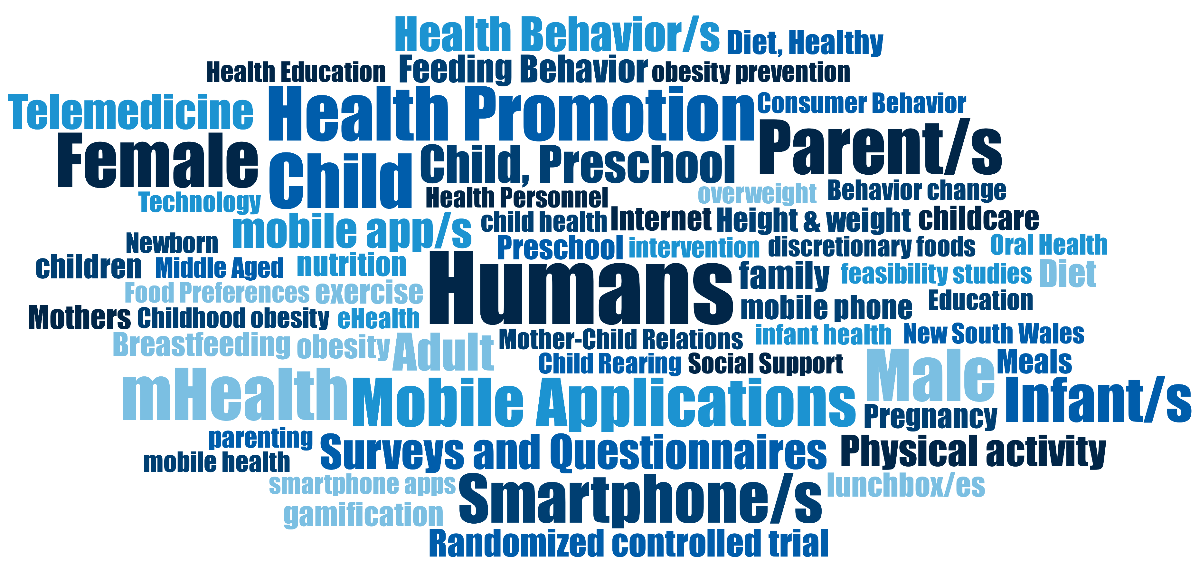

Supplement: Multimedia Appendix 3 [file mhealth_v11i1e39929_app3.png]

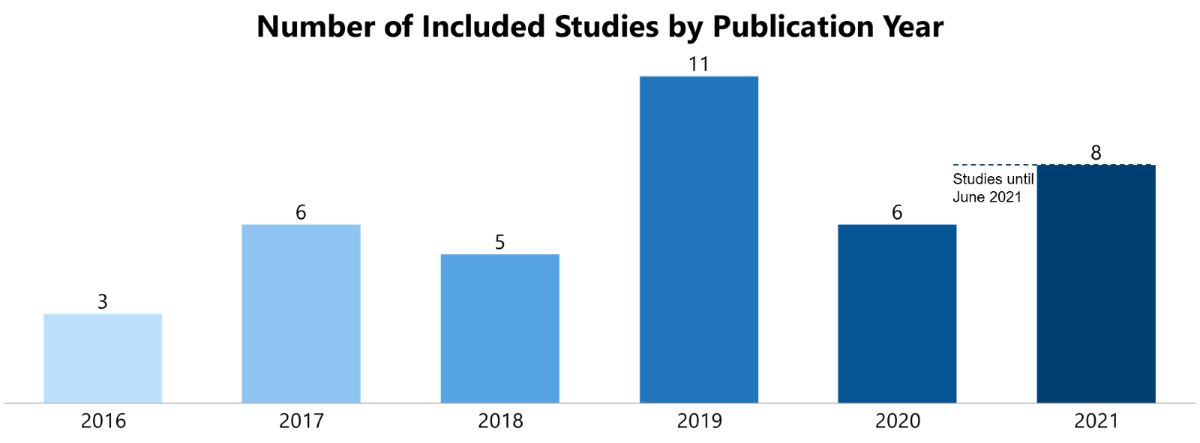

Supplement: Multimedia Appendix 4 [file mhealth_v11i1e39929_app4.png]
